# Activation of TLR2 and TLR6 by Dengue NS1 Protein and Its Implications in the Immunopathogenesis of Dengue Virus Infection

**DOI:** 10.1371/journal.ppat.1005053

**Published:** 2015-07-30

**Authors:** Jincheng Chen, Mary Mah-Lee Ng, Justin Jang Hann Chu

**Affiliations:** 1 Laboratory of Molecular RNA Virology and Antiviral Strategies, Department of Microbiology, Yong Loo Lin School of Medicine, National University Health System, National University of Singapore, Singapore; 2 Flavivirology Laboratory, Department of Microbiology, Yong Loo Lin School of Medicine, National University Health System, National University of Singapore, Singapore; Purdue University, UNITED STATES

## Abstract

Dengue virus (DV) infection is the most prevalent mosquito-borne viral disease and its manifestation has been shown to be contributed in part by the host immune responses. In this study, pathogen recognition receptors, Toll-like receptor (TLR) 2 and TLR6 were found to be up-regulated in DV-infected human PBMC using immunofluorescence staining, flow cytometry and Western blot analyses. Using ELISA, IL-6 and TNF-α, cytokines downstream of TLR2 and TLR6 signaling pathways were also found to be up-regulated in DV-infected PBMC. IL-6 and TNF-α production by PBMC were reduced when TLR2 and TLR6 were blocked using TLR2 and TLR6 neutralizing antibodies during DV infection. These results suggested that signaling pathways of TLR2 and TLR6 were activated during DV infection and its activation contributed to IL-6 and TNF-α production. DV NS1 protein was found to significantly increase the production of IL-6 and TNF-α when added to PBMC. The amount of IL-6 and TNF-α stimulated by DV NS1 protein was reduced when TLR2 and TLR6 were blocked, suggesting that DV NS1 protein is the viral protein responsible for the activation of TLR2 and TLR6 during DV infection. Secreted alkaline phosphatase (SEAP) reporter assay was used to further confirm activation of TLR2 and TLR6 by DV NS1 protein. In addition, DV-infected and DV NS1 protein-treated TLR6^-/-^ mice have higher survivability compared to DV-infected and DV NS1 protein-treated wild-type mice. Hence, activation of TLR6 via DV NS1 protein could potentially play an important role in the immunopathogenesis of DV infection.

## Author Summary

Despite the prevalence of dengue virus infection and the heavy economic burden it puts on the endemic countries, the immunopathogenesis of dengue virus infection remains unclear. Plasma leakage in dengue hemorrhagic fever (DHF) develops not when the viremia is at its peak in infected patients but when viremia has been significantly reduced or cleared. This suggests that host immune response is responsible for the development DHF. The interactions of the viral factors with host factors which trigger the host immune responses are likely to play a significant role in the development of dengue diseases, thus are of great interests. In this study, we found that dengue NS1 protein activates TLR2 and TLR6, leading to increase proinflammatory cytokine production. In addition, the interaction of viral factor with TLR6 was found to play an important role in the manifestation of dengue virus infection. Our study provides new insights into the involvement of TLR6 in dengue virus infection and the potential of using TLR6 anatagonist in therapeutic treatment for DV infection.

## Introduction

Dengue virus (DV) is a member of the *Flavivirus* genus of the *Flaviviridae* family. Dengue virus is a positive-sense, single-stranded RNA virus and it has four distinct serotypes (DV1 to 4). Infection by one serotype only confer resistance to the other serotypes for a few months and subsequent secondary infection of a different serotype has a higher risk of developing into the more severe forms of dengue infection; the dengue hemorrhagic fever or dengue shock syndrome [1–5]. Dengue virus genome encodes for a single polyprotein that consists of 3 structural proteins (capsid, premembrane and envelope) that form the physical structure of the virus particle and 7 non-structural proteins (NS1, NS2a, NS2b, NS3, NS4a, NS4b, NS5) which are necessary for the replication of the virus.

Dengue is a mosquito-borne viral disease transmitted through a human-to-mosquito-to-human transmission cycle typically by the *Aedes* mosquitoes: *Aedes aegypti* and *Aedes albopictus*. DV infection remains the most prevalent mosquito-borne viral disease and the geographical regions at risk are continually growing due to globalisation and climate change [6]. It is estimated that 100 million cases of dengue infection occur worldwide each year with 2.5 billion people at risk [7–9]. Till now, no effective treatment and vaccine are available for DV infection.

The pathogenesis of dengue is not well-understood. The mechanism underlying the wide range of dengue manifestations remain largely unknown. However, the observation that plasma leakage in DHF develops not when the viremia is at its peak in infected patients but when viremia has been significantly reduced or cleared, suggesting that host immune response is responsible for the development DHF [10–13]. In addition, studies have demonstrated that the host immunological mechanism could play a key role in the manifestation of dengue infection [3,14–16]. Up-regulation of proinflammatory cytokines and immune cells during dengue virus infection could lead to increased vascular permeability and leakage [17–20]. The hypotheses of antibody-dependent enhancement of infection and original antigenic sin have been proposed to explain the underlying mechanism that contributes to the manifestation of the more severe forms of the dengue infection during secondary infections [21–24].

Toll-like receptors (TLRs) are pathogen recognition receptors (PRRs). PRRs are a group of receptors that play a key role in immune surveillance. Pathogen recognition receptors are important as they alert the immune system of the presence of foreign microbes by recognizing pathogen-associated molecular patterns (PAMPs) and activating the immune system upon binding to PAMPs. In human, 10 functional TLRs are documented and each recognizing a group of PAMPs. When TLR is activated, adapter molecules like myeloid differentiaton primary response gene 88 (MyD88), toll-interleukin 1 receptor domain containing adaptor protein (Tirap), TIR-domain-containing adaptor-inducing interferon-β (Trif) and toll-like receptor 4 adaptor protein (Tram) are recruited. These adapter molecules in turn activate other downstream transcriptional gene regulators like activating protein-1, NFκB and interferon regulatory factors which induce expression of chemokines, proinflammatory cytokines [tumor necrosis factor alpha (TNF-α), IL-6, IL-1β and IL-12] or costimulatory molecules [25]. The up-regulation of costimulatory molecules is essential for the induction of pathogen-specific adaptive immune responses [26]. Thus, TLRs can activate both the innate and adaptive immune responses.

TLR can recognize viral pathogen via a number of different viral ligands. Generally, TLR3 detects double-stranded viral RNA, TLR2 and TLR4 sense the presence of virus via their proteins, TLR7/8 binds single-stranded viral RNA and TLR9 recognizes viral CpG DNA [27]. Among the TLRs, TLR3 and TLR7 have been found to trigger IL-8 production when stimulated by dengue viral RNA [28,29]. TLR6 was found to be up-regulated in DV2-infected K562 cells using Human Th1-Th2-Th3 RT^2^ Profiler PCR arrays in our previous study [30]. Although TLR6 was previously known only to be activated by diacylated lipoprotein of bacteria [31], recent studies have found that TLR6 can be activated by viruses including hepatitis C virus and respiratory syncytial virus [32,33]. Viral infection induces various TLR-mediated innate responses, which subsequently play a pivotal protective or pathogenic role in conjunction with virus-specific adaptive immune responses [34,35]. In the current study, dengue virus infection was found to activate and up-regulate TLR2 and TLR6 of human PBMC and DV NS1 protein was shown to be the viral protein responsible. Knockout of TLR6 increased the survivability of mice infected by dengue virus. Prolonged activation of TLR6 by DV NS1 protein during DV infection could be responsible for the lower survivability observed in wild-type mice compared to the TLR6^-/-^ mice. Hence, TLR6 may play an important role in the immunopathogenesis of dengue virus infection.

## Results

### 1) Dengue virus induces activation and up-regulation of TLR2 and TLR6 which leads to higher production of IL-6 and TNF-α by PBMC

In our previous study [30], several genes involved in the TLR6 pathway have been found to be significantly up-regulated during dengue virus infection in K562 cells on day 3 post-infection which include TLR6, IL-6, TNF-α and CD80. TLR6 pathway activation is well-documented to play an important part in activating both the innate and adaptive immunity. Human PBMC were found to express the whole range of human TLRs (TLR1-10) [36]. The expression of TLRs has been found to increase following inflammations and exposure to pathogens or specific ligands [37–40].

**Fig 1:**
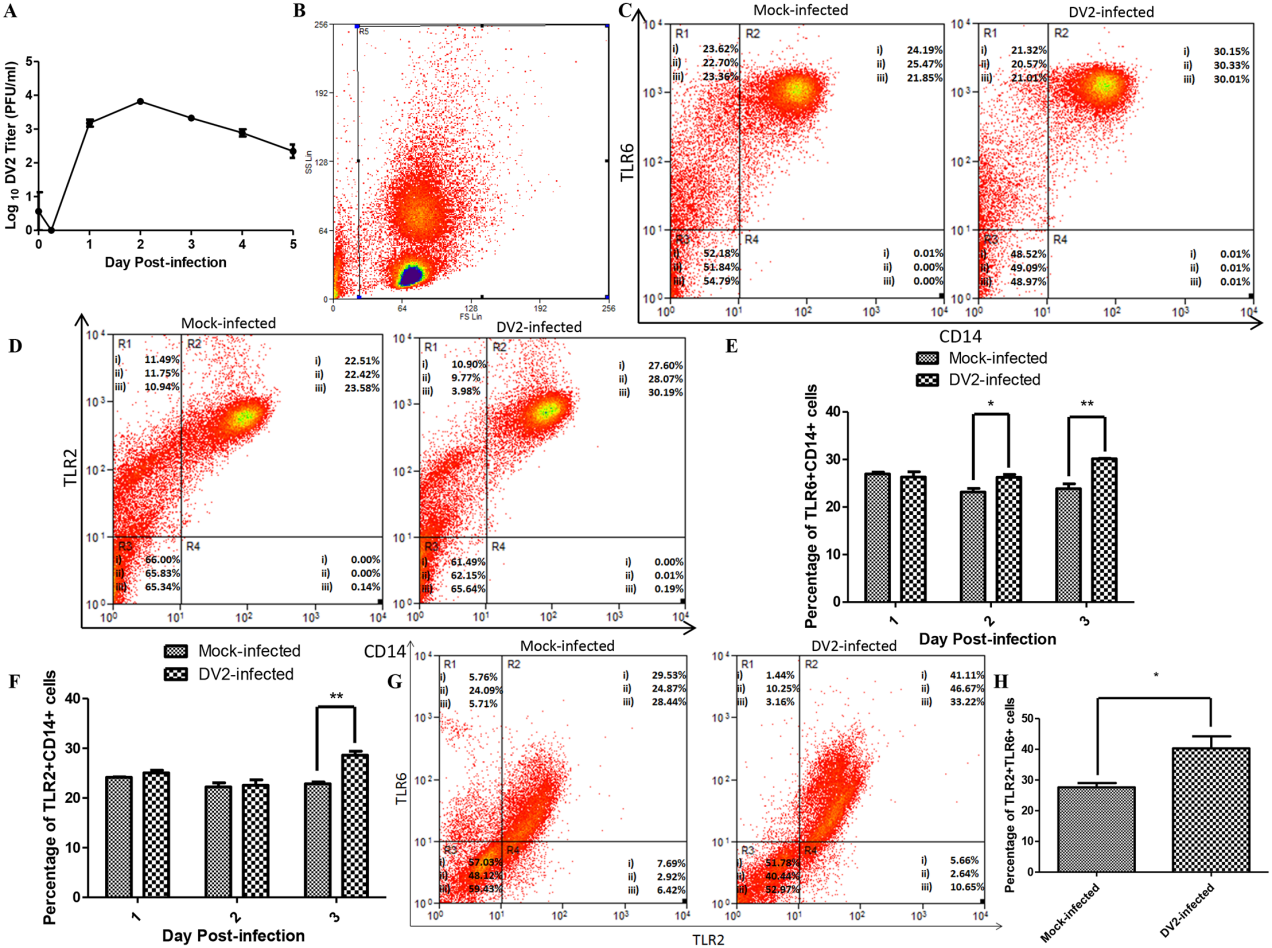
Expression of TLR2 and TLR6 of DV2-infected PBMC. (A) Growth curve of DV2 in PBMC was determined using plaque assays (0 hr, 6 hrs, 1 to 5 days). Values plotted are mean ± SEM of three independent experiments performed in triplicates obtained using separate PBMC from three donors. Mock-infected and DV2-infected PBMC on day 3 post-infection were stained using rabbit anti-TLR6 antibody, goat anti-rabbit DyLight 633 (APC) antibody and mouse anti-CD14 antibody conjugated with FITC. The cell debris was excluded by gating as shown in (B) and the stained cells were analyzed using flow cytometry (C). The percentages of cell subset population were indicated on the representative results of three independent experiments obtained using separate PBMC from three donors (i, ii & iii). Data for day 1 and day 2 post-infection were shown in S1 Fig. The percentages of TLR6+CD14+ cells of mock-infected and DV2-infected PBMC were plotted on a graph (E). Mock-infected and DV2-infected PBMC on day 3 post-infection were also stained using mouse anti-TLR2 antibody, goat anti-mouse DyLight 633 (APC) antibody and mouse anti-CD14 antibody conjugated with FITC. The cell debris was excluded by gating as shown in (B) and the stained cells were analyzed using flow cytometry (D). The percentages of cell subset population were indicated on the representative results of three independent experiments obtained using separate PBMC from three donors (i, ii & iii). Data for day 1 and day 2 post-infection were shown in S2 Fig. The percentages of TLR2+CD14+ cells of mock-infected and DV2-infected PBMC were plotted on a graph (F). The PBMC from three donors (i, ii & iii) were harvested on day 3 post-infection and stained using rabbit anti-TLR6 antibody, goat anti-rabbit DyLight 633 (APC) antibody and mouse anti-TLR2 antibody conjugated with FITC (G). 30, 000 cells were analyzed and gating was set to remove cell debris. Region 1 (R1) contains cells which are negative for TLR2 but positive for TLR6. Region 2 (R2) contains cells which are positive for both TLR2 and TLR6. Region 3 (R3) contains cells which are negative for both TLR2 and TLR6. Region 4 (R4) contains cells which are positive for TLR2 but negative for TLR6. The percentages of TLR2+TLR6+ cells of mock-infected and DV2-infected PBMC were plotted on a graph (H).

The susceptibility of the PBMC to DV2 infection was first determined using plaque assay (Fig 1A). The virus titer peaked on day 2 post-infection (3.82 Log_10_PFU/ml) and decreases from day 3 to day 5 post-infection. The increase in virus titer on day 2 post-infection of PBMC provided evidence of replication of DV2 in PBMC.

Next, flow cytometric analyses were performed to determine the expression of TLR6 of mock-infected and DV2-infected PBMC. Gating was used to exclude the cell debris (Fig 1B). PBMC were also stained with anti-CD14 FITC conjugated antibody to serve as a marker for human monocytes [41,42]. Monocytes are the main cells in PBMC that express TLR2 and TLR6 **[** 31]. Upon dengue virus infection, higher percentages of TLR6+CD14+ cell population was observed compared to the mock-infected PBMC on day 2 and 3 post-infection (Figs 1C, 1E, and S1). TLR6 requires heterodimerization with TLR2 to recognize ligand and trigger cytokine production [43–45]. Hence, TLR2 expression was also investigated. TLR2+CD14+ cell population was up-regulated upon DV infection on day 3 post-infection (Figs 1D, 1F, and S2). CD14+ monocytes expressing TLR2 were reported by Azeredo and colleagues (2010) to be increased in peripheral blood of dengue patients. In addition, DV2-infected PBMC expressed higher level of TLR6 and TLR2 on day 3 post-infection but not day 1 and day 2 post-infection (S3 Fig).

PBMC were also stained for both TLR2 and TLR6 simultaneously for flow cytometric analysis. In addition, the CD3 and CD20 coexpression on PBMC were analyzed as high percentage of CD3+CD20+ cell population could suggest neoplastic transformation. The percentage of PBMC expressing both CD3 and CD20 were 3.84% and 3.94% which are within the range detected by other research groups using healthy donors [46–48]. The percentages of mock-infected PBMC which were TLR2+ and TLR6+ were lower than the percentages of DV2-infected PBMC which were TLR2+ and TLR6+ (Fig 1G, 1H and S1 Table). The median fluorescence intensity of the TLR2 and TLR6 were also higher for the DV2-infected than the mock-infected (S1 Table).

**Fig 2:**
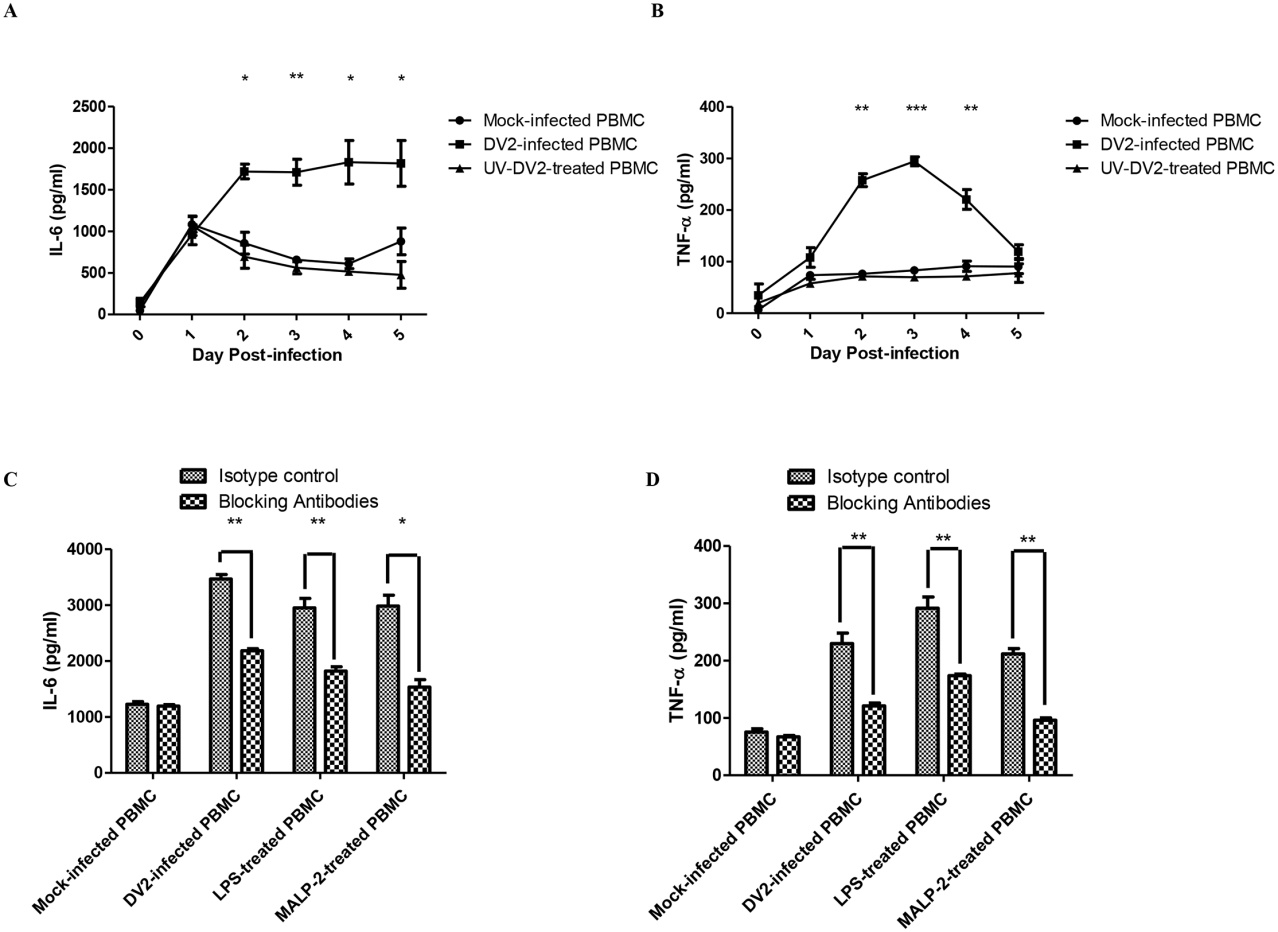
Activation of TLR2 and TLR6 of DV2-infected PBMC. ELISA was performed to quantify the amount of IL-6 (A) and TNF-α (B) secreted into the supernatant by mock-infected and DV2-infected PBMC. Data represent mean ± SEM of three independent experiments obtained using separate PBMC from three donors. Student’s *t-*tests were used to compare mean amount of IL-6/TNF-α detected in culture supernatants of DV2-infected PBMC to that of the mock-infected PBMC. ELISA was also performed to quantify the amount of IL-6 (C) and TNF-α (D) secreted into the supernatant by mock-infected, DV2-infected, LPS-treated and MALP-2 treated PBMC on day 2 post-treatment. Data represent mean ± SEM of three independent experiments obtained using separate PBMC from three donors. Student’s *t-*tests were used to compare mean amount of IL-6/TNF-α detected in culture supernatants of TLR2 and TLR6 blocked PBMC to that of the isotype control PBMC.

After affirming the up-regulation of TLR2 and TLR6 of PBMC when infected by DV, activation of these receptors during DV infection were investigated by measuring the amount of IL-6 secreted into the extracellular milieu by the DV2-infected PBMC. The amount of IL-6 in the culture media of DV2-infected PBMC increased significantly from day 2 to day 5 post-infection as compared to that of the mock-infected PBMC (Fig 2A). Similarly, the DV2-infected PBMC significantly up-regulated the amount of TNF-α secreted into the extracellular mileu from day 2 to day 4 post-infection, compared to that of the mock-infected PBMC (Fig 2B). UV-inactivated DV did not induce up-regulation of IL-6 (Fig 2A) and TNF-α (Fig 2B) when added to PBMC culture. This could suggest that viral replication is required for the up-regulation of IL-6 and TNF-α. To determine if the up-regulation of IL-6 and TNF-α detected were contributed by TLR2 and TLR6 activation, TLR2 and TLR6 blocking antibodies were used. Blocking of TLR2 and TLR6 reduced the amount of IL-6 produced by PBMC when stimulated by LPS (5 μg/ml) or MALP-2 (50 ng/ml), compared to that of the isotype control (Fig 2C). MALP-2 is a 2-kDa synthetic derivative of the macrophage-activating lipopeptide and it is a specific agonist for TLR2 andTLR6. Blocking of TLR2 and TLR6 also reduced the amount of IL-6 produced by PBMC during dengue virus infection, this suggested that TLR2 and TLR6 are activated during dengue virus infection and this activation led to increase in IL-6 secretion. Similar observation was made for TNF-α production by PBMC (Fig 2D). This suggested that TLR2 and TLR6 are the receptors activated during DV infection to result in the increase in IL-6 and TNF-α expression.

### 2) DV NS1 protein is the viral protein responsible for the activation and up-regulation of TLR2 and TLR6

**Fig 3:**
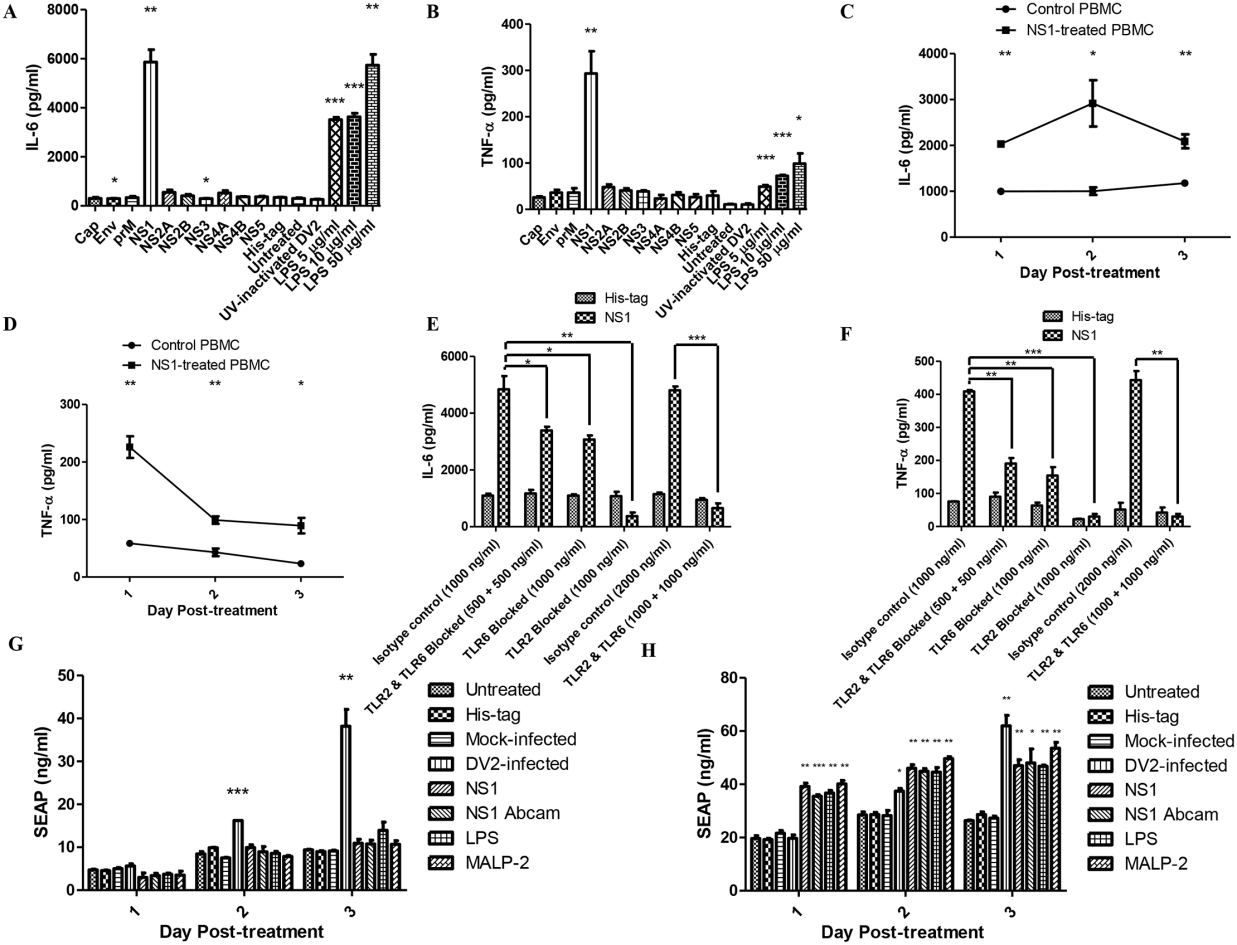
DV NS1 protein activates TLR2 and TLR6 of PBMC. The amount of IL-6 (A) and TNF-α (B) secreted into the supernatant by PBMC treated with dengue viral proteins (capsid, envelope, premembrane, NS1, NS2A, NS2B, NS3, NS4A, NS4B, NS5, each 50 μg/ml), His-tag-treated PBMC, untreated PBMC, UV-inactivated DV2-treated PBMC (10 virus particles per cell), LPS-treated PBMC (5 μg/ml, 10 μg/ml, 50 μg/ml) on day 2 post-treatment were quantified using ELISA. Data represent mean ± SEM of three independent experiments obtained using separate PBMC from three donors. Student’s *t-*tests were used to compare mean amount of IL-6/TNF-α detected in culture supernatants of viral protein-treated PBMC to that of the His-tag-treated PBMC, the mean amount of IL-6/TNF-α detected in culture supernatants of LPS-treated PBMC to that of the untreated PBMC, the mean amount of IL-6/TNF-α detected in the culture supernatants of His-tag-treated PBMC to that of the untreated PBMC and the mean amount of IL-6/TNF-α detected in the culture supernatants of UV-inactivated DV2-treated PBMC to that of the untreated PBMC. The amount of IL-6 (C) and TNF-α (D) secreted into the supernatant by DV NS1 protein-treated PBMC (1 μg/ml) on day 1 to day 3 post-treatment were quantified using ELISA. Data represent mean ± SEM of three independent experiments. Student’s *t-*tests were used to compare mean amount of IL-6/TNF-α detected in culture supernatants of DV NS1 protein-treated PBMC to that of the His-tag-treated PBMC. The amount of IL-6 (E) and TNF-α (F) secreted into the supernatant by DV NS1 protein-treated PBMC (1 μg/ml) on day 1 post-treatment were quantified using ELISA. TLR2 and TLR6 of the PBMC were blocked prior to the treatment. Data represent mean ± SEM of three independent experiments obtained using separate PBMC from three donors. Student’s *t-*tests were used to compare mean amount of IL-6 detected in culture supernatants of DV NS1 protein-treated PBMC isotype control to that of DV NS1 protein-treated TLR2/6 blocked PBMC. HEK 293 cells transfected with SEAP reporter plasmid (G), HEK 293 cells transfected with both SEAP reporter plasmid and pDUO-hTLR6/TLR2 plasmid (H) were treated with His-tag (1 μg/ml), DVNS1 recombinant protein (1 μg/ml), LPS (5 μg/ml), MALP-2 (50 ng/ml), mock-infected or DV2-infected (M.O.I of 10). The supernatants of the HEK 293 cell cultures were harvested on day 1 to day 3 post-treatment. The SEAP in the supernatants were quantified using SEAP reporter assay kit. Data represent mean ± SEM of three independent experiments. Student’s *t-*tests were used to compare mean amount of SEAP detected in culture supernatants of DV NS1 protein-treated HEK 293 cells to that of His-tag-treated HEK 293 cells, mock-infected HEK 293 cells to that of DV2-infected HEK 293 cells, LPS-treated HEK 293 cells to that of untreated and MALP-2 HEK 293 cells to that of untreated HEK293 cells.

To determine if any specific viral protein is responsible for the activation of TLR2 and TLR6, IL-6 and TNF-α expression by PBMC after treatments with individual viral proteins were assayed. The detection of up-regulation of IL-6 and TNF-α by PBMC would indicate possible activation of receptors by the dengue viral proteins. ELISA was performed to quantify the amount of IL-6 secreted into the supernatant by PBMC after treatment with the individual dengue viral proteins on day 2 post-treatment. Among the dengue viral proteins, DV NS1 protein is the only viral protein which stimulated high amount of IL-6 expression (5864 pg/ml) (Fig 3A). IL-6 expression was slightly down-regulated by dengue envelope protein (296 pg/ml) and NS3 protein (297 pg/ml) compared to His-tag-treated PBMC (346 pg/ml). The IL-6 level of His-tag-treated PBMC was comparable to that of the untreated, suggesting that His-tag did not trigger IL-6 production and the IL-6 detected in the culture supernatant of the His-tag-treated PBMC was due to basal expression. The IL-6 level of UV-inactivated DV2-treated PBMC was also comparable to that of the untreated. This suggested that non-replicative virus cannot induce IL-6 expression. The positive control, LPS was found to stimulate IL-6 production by PBMC. ELISA was also performed to determine which of the viral protein can induce up-regulation of TNF-α by PBMC. DV NS1 protein is the only viral protein which stimulated high amount of TNF-α expression (293 pg/ml) compared to that of the His-tag-treated PBMC (30 pg/ml) (Fig 3B). Similar to IL-6, the TNF-α level of His-tag-treated PBMC was comparable to that of the untreated and the TNF-α level of UV-inactivated DV2-treated PBMC was also comparable to the untreated. The positive control, LPS was also found to stimulate TNF-α production by PBMC. The result suggested that DV NS1 protein is the viral protein that stimulates IL-6 and TNF-α production by PBMC during DV infection.

Next, lower concentration of DV NS1 protein (1 μg/ml) was used to stimulate PBMC. This concentration of DV NS1 protein is within the concentration range detected in dengue patients (several nanograms per millilitre to several micrograms per millilitre) [49]. ELISA was performed to quantify the amount of IL-6 in the supernatants of DV NS1-treated PBMC on day 1 to day 3 post-treatment. DV NS1 protein-treated PBMC produced significantly higher amount of IL-6 compared to that of the His-tag-treated PBMC from day 1 post-treatment (Fig 3C). The up-regulation was faster than that of the DV2-infected PBMC which only produced significantly higher IL-6 from day 2 post-treatment (Fig 2A). The delay observed in the DV2-infected PBMC could be due to time required for DV NS1 protein synthesis during DV replication and secretion. The secreted DV NS1 protein can then be detected by the cell surface receptors, TLR2 and TLR6. The IL-6 produced by DV NS1 protein-treated PBMC peaked on day 2 post-treatment (2917 pg/ml).

Similarly, the amount of TNF-α produced by DV NS1 protein-treated PBMC was quantified using ELISA. DV NS1 protein-treated PBMC produced significantly higher amount of TNF-α compared to that of the His-tag-treated PBMC from day 1 post-treatment (Fig 3D). The amount of TNF-α produced by DV NS1 protein-treated PBMC peaked on day 1 post-treatment (226 pg/ml). The amount of TNF-α decreased from day 2 to day 3. To determine if the IL-6 production by PBMC upon DV NS1 protein stimulation is through TLR2 and TLR6, TLR2 and TLR6 neutralizing antibodies were used. The specificity of TLR2 and TLR6 blocking antibodies were tested using TLR4 specific ligand, ultrapure LPS (S4 Fig). TLR2 and TLR6 blocking antibodies did not affect TLR4. TLR2 and TLR6 of PBMC were blocked by the neutralizing antibodies prior to addition of DV NS1 protein into the PBMC culture (1 μg/ml). The supernatant of the treated PBMC were collected on day 1 post-treatment and IL-6 was quantified using ELISA. Day 1 was chosen as the time point as IL-6 was found to be significantly up-regulated from day 1 post-treatment in Fig 3C. With both TLR2 and TLR6 blocked, IL-6 secreted by DV NS1 protein-treated PBMC was significantly reduced compared to that of the isotype control (Fig 3E). With only TLR6 blocked, IL-6 secreted by DV NS1 protein-treated PBMC was comparable to that of both TLR2 and TLR6 blocked (Fig 3E). Therefore, DV NS1 protein stimulation of IL-6 requires both TLR2 and TLR6. The stimulation is inhibited when one of the receptors is blocked. With only TLR2 blocked, IL-6 secreted by DV NS1 protein-treated PBMC was significantly reduced and surprisingly lower than that of the His-tag-treated PBMC (Fig 3E). High amount of TLR2 neutralizing antibody may have some effect on the basal IL-6 expression of PBMC. TLR2 neutralizing antibody was found to be more effective than TLR6 neutralizing antibody. The expected level of IL-6/TNF-α for DV NS1 protein-treated PBMC with 500 ng/ml of TLR2 and 500 ng/ml of TLR6 neutralizing antibodies should be between the level of IL-6/TNF-α for DV NS1 protein-treated PBMC with 1000 ng/ml of TLR2 only and those with 1000 ng/ml of TLR6 only. The presence of the more efficient TLR2 blocking antibodies in the treatment group with both blocking antibodies should be able to more efficiently block the TLR2/6 pathway compared with the treatment with only TLR6 blocking antibody. However, the observation was not the case. The expected result would only happen if the TLR2 and TLR6 antibodies can sterically hinder the binding of each other to prevent one TLR2/6 complex from binding both TLR2 and TLR6 antibodies at the same time. The observed result suggested that the two antibodies did not sterically hinder each other. Thus, one TLR2 and one TLR6 antibodies can bind and inhibit the same TLR2/6 complex, an inhibition which can be achieved initially with just either one TLR antibody.

In summary, DV NS1 protein-treated PBMC which were also treated with TLR2 or TLR6 neutralizing antibodies or both secreted less IL-6 compared to the isotype control. Together, these data implied that TLR2 and TLR6 are the receptors activated by DV NS1 protein. Similar to what was observed for IL-6, the TNF-α amount secreted by His-tag-treated PBMC in general, was not affected by the neutralizing antibodies (Fig 3F). With both TLR2 and TLR6 blocked, TNF-α secreted by DV NS1 protein-treated PBMC was significantly reduced compared to that of the isotype control. With only TLR6 blocked, TNF-α secreted by DV NS1 protein-treated PBMC was comparable to that of both TLR2 and TLR6 blocked. With only TLR2 blocked, TNF-α secreted by DV NS1 protein-treated PBMC was significantly reduced. With 1000 ng/ml of TLR2 neutralizing antibody, the basal TNF-α expression of His-tag-treated PBMC was affected, as shown by the lower TNF-α level of the TLR2 blocked His-tag-treated PBMC compared to that of the isotype control His-tag-treated PBMC. This may suggest that TLR2 activation partially contributed to the basal expression of IL-6 and TNF-α detected in our PBMC culture. In summary, PBMC which were treated with TLR2 and/or TLR6 neutralizing antibodies secreted less TNF-α compared to the isotype control. Together, the data implied that TLR2 and TLR6 are the receptors activated by DV NS1 protein.

In addition, SEAP reporter assay was used to further confirm if DV NS1 protein is activating TLR2 and TLR6 using the HEK 293 cells. HEK 293 cell line which naturally does not possess many of the TLRs was also used for the reporter assay. HEK 293 cells have good transfection efficiency to allow expression of desired TLR and the SEAP reporter plasmid for investigating specific TLR ligand. HEK 293 cells were found to express low level of endogenous TLR6 but not TLR2 [50,51]. The reports of low level of expression of TLR6 and no expression of TLR2 in HEK 293 cells were further confirmed in our western blot results (S5 Fig). LPS and MALP-2 were used as positive control. For HEK 293 cells transfected with only SEAP reporter plasmid, the SEAP secretion by HEK 293 cells treated with DV NS1 protein and the positive controls were not significantly different from the negative controls (untreated and His-tag-treated HEK 293 cells) (Fig 3G). This suggested that DV NS1 protein, LPS and MALP-2 cannot stimulate NFκB activation in the absence of TLR2. DV2-infected HEK 293 cells produced significantly higher SEAP than mock-infected HEK 293 cells. This suggested that DV2-infection can trigger NFκB activation through pathway independent of TLR2. For HEK 293 cells transfected with SEAP reporter, TLR2 and TLR6 expression plasmids, the SEAP secretion by HEK 293 cells treated with DV NS1 protein and the positive controls were significantly different from the negative controls from day 1 post-treatment (Fig 3H). The result suggested that activation of NFκB by DV NS1 protein is dependent on both TLR2 and TLR6.

**Fig 4:**
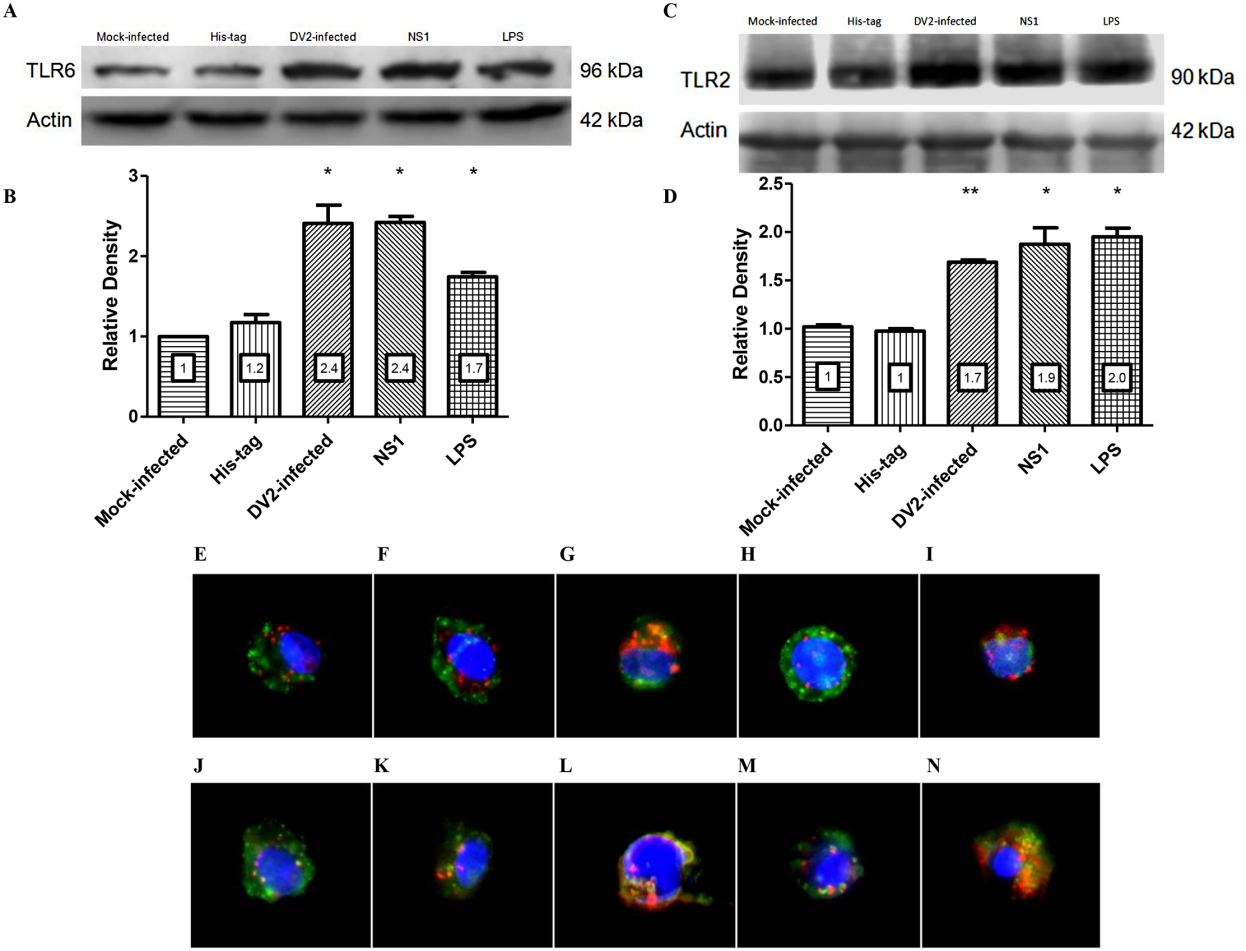
DV NS1 protein up-regulates TLR2 and TLR6 of PBMC. Mock-infected, His-tag-treated, DV2-infected, DV NS1 protein-treated, LPS-treated PBMC were lysed and equal total protein was loaded into SDS-PAGE gel. PBMC of two donors were used. The presence of TLR6 protein in the cell lysate was detected using rabbit anti-TLR6 antibody and goat anti-rabbit HRP conjugated antibody on the Western blot (A). Actin was used as loading control. The band intensity was measured using GelQuantNET software program. The intensities of the TLR6 bands were normalized against the intensity of the corresponding actin bands and were plotted in the graph (B). The presence of TLR2 protein in the cell lysate was detected using rabbit anti-TLR2 antibody and goat anti-rabbit HRP conjugated antibody on the Western blot (C). Actin was used as loading control. The intensities of the TLR2 bands were normalized against the intensity of the corresponding actin bands and were plotted in the graph (D). The relative intensities of the bands were indicated on the charts. PBMC were untreated (E & J), mock-infected (F & K), DV2-infected (G & L), His-tag-treated (H & M) and DV NS1 protein-treated (I & N). The PBMC were harvested at day 3 post-treatment. TLR6 was stained red using primary rabbit anti-TLR6 antibody and secondary goat anti-rabbit DyLight 633 antibody (E to I). TLR2 is stained red using primary mouse anti-TLR2 antibody and secondary goat anti-mouse DyLight 633 (J to N). CD14 was stained green using anti-CD14 antibody conjugated with FITC (E to N). Cell nuclei are stained blue with DAPI. Representative images from triplicate experiments obtained using separate PBMC from three donors are shown.

DV2-infection was found to up-regulate TLR6 in PBMC (Fig 1E and 1H). To determine if this up-regulation is contributed by DV NS1 protein effect on the cells, Western blot analyses were used. PBMC were treated with His-tag (1 μg/ml), DV NS1 protein (1 μg/ml), LPS (5 μg/ml), mock-infected or DV2-infected (M.O.I of 10). TLR6 bands of DV2-infected, DV NS1 protein-treated and LPS-treated PBMC were of higher intensity than those of mock-infected and His-tag-treated PBMC (Fig 4A). The relative density of the TLR6 bands normalized against the actin bands was plotted on a graph (Fig 4B). The result suggested that DV NS1 protein can stimulate up-regulation of TLR6 in PBMC. Similarly, TLR2 bands of DV2-infected, DV NS1 protein-treated and LPS-treated PBMC were of higher intensity than those of mock-infected and His-tag-treated PBMC (Fig 4C). The relative density of the TLR2 bands normalized against the actin bands was plotted on a graph (Fig 4D). The results suggested that DV NS1 protein can stimulate up-regulation of TLR2 and TLR6 in PBMC.

TLR2 and TLR6 expression on PBMC were further investigated using immunofluorescence analyses. Mock-infected and DV2-infected PBMC were harvested on day 3 post-infection and stained for TLR2/TLR6 and CD14. The staining of untreated PBMC (Fig 4E and 4J) were comparable to that of the mock-infected (Fig 4F & 4K) and His-tag-treated PBMC (Fig 4H and 4M). Similar to the results obtained in flow cytometric and western blot analyses, TLR2/TLR6 was up-regulated in DV2-infected PBMC (Fig 4G and 4L), indicated by the denser red spots compared to the mock-infected PBMC (Fig 4F and 4K). TLR2/TLR6 was also up-regulated in DV NS1 protein-treated PBMC (Fig 4I and 4N) compared to the His-tag-treated PBMC (Fig 4H and 4M). Colocalization of both TLR2 and TLR6 with CD14 were observed for untreated, mock-infected, DV2-infected, His-tag-treated and DV NS1 protein-treated PBMC (yellow stains). Hence, the colocalization of the receptors could be independent of infection or DV NS1 protein stimulation. It was reported that TLR2 and TLR6 heterodimers pre-exist and are not induced by ligand [45].

### 3) Knockout of TLR6 increases the survivability of DV2-infected mice using dengue murine model

**Fig 5:**
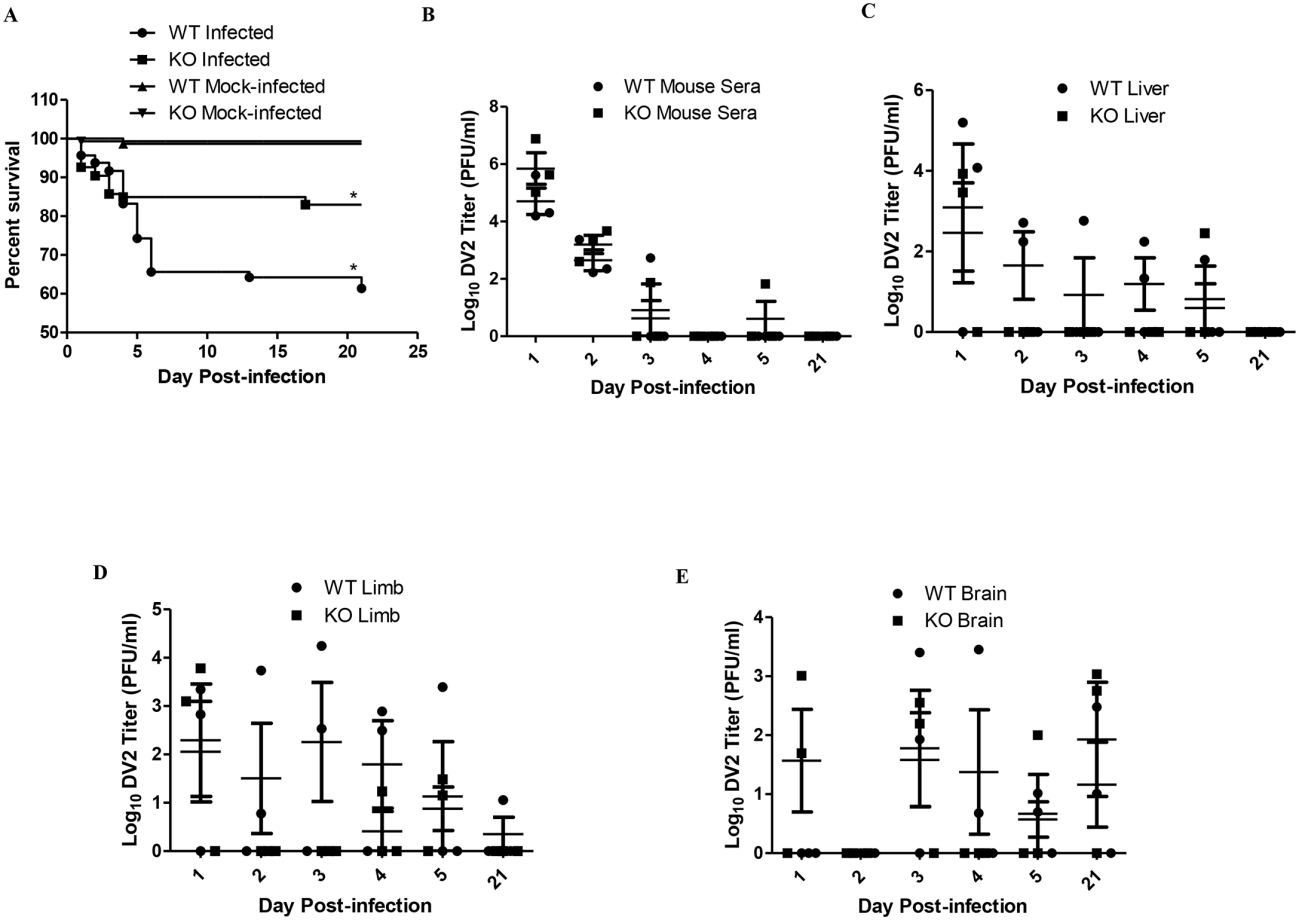
TLR6 knockout enhanced the survivability of DV2-infected mice. (A) Wild-type (184 mice) and TLR6^*-/-*^ (203) mice were infected with 2.7 x 10^8^ PFU of DV2 strain 16681 at 1–2 day-old via intraperitoneal injection. Mock-infected wild-type (140 mice) and TLR6^*-/-*^ (146) mice were injected with 50 μl of C6/36 cell culture supernatant. The survival of the mice was monitored from day 1 to day 21 on a daily basis. Kaplan-Meier survival curves were shown. Log-rank test was performed to determine if there was significant difference in survivability between DV2-infected wild-type and TLR6^*-/-*^ mice. The blood and tissues of mice were harvested on day 1, 2, 3, 4, 5 and 21 post-infection via cardiac puncture. The virus in the sera (B), livers (C), limbs (D) and brains (E) of mice were quantified by plaque assays. Virus titers of 0 PFU/ml were set to 0 Log10 PFU/ml to be reflected on the log scale graph. No actual virus titer of 0 Log10 PFU/ml was obtained from any of the samples. Each sample group is in triplicates. Each set of samples was in triplicates.

The activation of TLR2 and TLR6 could be a double-edged sword that is both beneficial and detrimental to the host. To assess the potential role of the activation of TLR2 and TLR6 plays during dengue virus infection, the use of cell model is not sufficient, an animal model is required. Wild-type and TLR6 knockout (TLR6^*-/-*^) C57BL/6 mice were used in this part of the study. In order to determine if TLR6 activation during dengue virus infection contributes to the pathogenesis of the disease, wild-type and TLR6^*-/-*^ mice were injected with 2.7 x 10^8^ PFU of DV2 on day 1–2 day-old (Fig 5A). The survival rate of the DV2-infected wild-type mice was 61.4% at the end point of the study. The survival rate of the TLR6^*-/-*^ DV2-infected mice was 83.0% at the end point of the study. Knockout of TLR6 increased the survival rate of the mice at the end point of the study by 21.6%, suggesting that activation of TLR6 may contribute to the pathogenesis of the disease, leading to higher fatality observed in the DV2-infected wild-type mouse population. Using Log-rank test, DV2-infected wild-type mice survival curve was found to be statistically different from DV2-infected TLR6^*-/-*^ mice. Hence, knockout of TLR6 significantly enhanced the survival rate of the DV2-infected mice.

Next, we investigated what could have resulted in the difference in survival rate of wild-type and TLR6^*-/-*^ mice. Pups which were 1–2 day-old were injected with 2.7 x 10^8^ PFU of DV2 and quantified for virus titer in the sera and livers. DV2 were detected in all the DV2-infected pups from day 1 to day 2 post-infection. The average virus titer detected in the sera of the DV2-infected wild-type mice on day 1 was 1.51 x 10^5^ PFU/ml while that on day 2 was 9.17 x 10^2^ PFU/ml and that on day 3 was 1.81 x 10^2^ PFU/ml (Fig 5B and Table 1). This suggested that the pups were susceptible to dengue virus infection. 1–2 day-old TLR6^*-/-*^ mice were also infected in the same way as the wild-type. Virus in the sera of TLR6^*-/-*^ mice was also quantified. The average virus titer detected in the sera of the DV2-infected TLR6^*-/-*^ mice was 2.73 x 10^6^ PFU/ml on day 1 while that on day 2 was 2.40 x 10^3^ PFU/ml and that on day 3 was 2.54 x 10^1^ PFU/ml (Fig 5B). Comparing the virus titers obtained in the sera of DV2-infected wild-type and TLR6^*-/-*^ mice, virus titers were not statistically significantly different. This suggested similar susceptibility of wild-type and TLR6^*-/-*^ mice to DV2. Viremia persisted in both wild-type and TLR6^*-/-*^ mice till day 3. By day 4 post-infection, virus can no longer be detected in the sera of mice except for one TLR6^*-/-*^ mice whose serum detected presence of DV2 on day 5 post-infection. Virus titers in the livers of wild-type and TLR6^*-/-*^ mice were also quantified using plaque assay (Fig 5C). Unlike what was observed for the sera, DV2 was not detected in every liver of the DV2-infected mice on day 1 and 2 post-infection. On the contrary, DV2 were detected in the livers of DV2-infected mice more frequently on day 4 and day 5 post-infection compared to that of the sera. This may suggest that though not all the DV2 can establish infection in the liver organ, for those that established, it can persist longer in the liver than in the sera. The sera and livers of both the wild-type and TLR6^*-/-*^ mock-infected mice were detected to be absent of DV2.

**Table 1:** Virus titers of DV2 in sera of DV2-infected wild-type and TLR6 KO mice.

Day Post-infection	DV2-Infected Wild-type (PFU/ml)			DV2-Infected TLR6 KO (PFU/ml)		
**1**	**15625**	**416667**	**20000**	**426667**	**104902**	**7666674**
**2**	**167**	**2361.11**	**222.222**	**400**	**4696.97**	**2111.11**
**3**	**540**	**0**	**0**	**74.074**	**0**	**0**
**4**	**0**	**0**	**0**	**0**	**0**	**0**
**5**	**0**	**0**	**0**	**66.6667**	**0**	**0**
**21**	**0**	**0**	**0**	**0**	**0**	**0**

DV2-infected mice developed some abnormal signs like enlarged belly, hind limb paralysis, moribundity and death (Table 2). Hindlimb paralysis was also observed in DV2-infected mice. Symptoms of paralysis of extremities which has been observed in some dengue patients were also observed in the murine model [52]. However, such occurrences were rare. From the observations of the mice on a daily basis, the occurrence of paralysis was observed on day 10–14 post-infection.

**Table 2:** Virus titers of DV2 in symptomatic mice. Mice which were observed to have developed certain symptoms of infection were noted. Besides harvesting sera, brains, livers and limbs of the symptomatic mice, abnormal tissue (intestine) or peritoneal fluid discovered were also harvested to be quantified for DV2 virus titer.

Symptom	Sample	Day post-infection	Virus Titer Serum/Fluid: PFU/ml Liver/Brain/Limb: PFU/g
Death	WT Brain	4	0
Death	WT Brain	4	0
Death	WT Liver	4	0
Death	WT Limb	4	0
Death	WT Peritoneal fluid	1	1.11 x 10^4^
Moribundity	WT Brain	6	3.07 x 10^5^
Moribundity	WT Liver	6	6.31 x 10^3^
Moribundity	WT Limb	6	2.01 x 10^5^
Moribundity	WT Serum	5	0
Moribundity	WT Serum	5	0
Hindlimb paralysis	WT Brain	10	2.76 x 10^4^
Hindlimb paralysis	WT Brain	11	5.24 x 10^5^
Hindlimb paralysis	WT Brain	14	8.16 x 10^5^
Hindlimb paralysis	WT Liver	14	0
Hindlimb paralysis	WT Liver	11	0
Hindlimb paralysis	WT Liver	10	0
Hindlimb paralysis	WT Limb	14	9.49 x 10^1^
Hindlimb paralysis	WT Limb	11	1.38 x 10^3^
Hindlimb paralysis	WT Limb (unparalyzed)	11	4.01 x 10^3^
Hindlimb paralysis	WT Serum	12	0
Hindlimb paralysis	WT Serum	12	0
Hindlimb paralysis	KO Brain	13	7.10 x 10^5^
Hindlimb paralysis	KO Liver	13	0
Hindlimb paralysis	KO Limb	13	0
Hindlimb paralysis	KO Serum	13	0
Hindlimb paralysis & Recovered	KO Liver	14	0
Hindlimb paralysis & Recovered	KO Brain	14	2.12 x 10^4^
Hindlimb paralysis & Recovered	KO Limb	14	0
Hindlimb paralysis & Swollen intestine	WT Intestine	14	4.27 x 10^2^
Bulging belly	WT Liver	3	0
Bulging belly	WT Serum	1	1.36 x 10^5^
Bulging belly	WT Serum	1	3.00 x 10^5^
Bulging belly	WT Peritoneal fluid	1	1.13 x 10^6^
Bulging belly	WT Peritoneal fluid	1	1.27 x 10^6^
Bulging belly	KO Brain	2	0
Bulging belly & Death	KO Peritoneal fluid	2	3.50 x 10^3^
Bulging belly & Death	KO Liver	2	0
Bulging belly & Death	KO Limb	2	0

Some of the DV2-infected mice were found to succumb to the infection. No viable virus was detected in the tissues of the dead mice which could be due to decomposition. Blood of the dead mice could not be harvested due to the clotting of the blood. High virus titers were detected in the brain, liver and limbs of the moribund mice, the virus titers were higher than the average virus titers detected in the brain, liver and limbs of the asymptomatic DV2-infected mice (Table 2). However, no virus was detected in the sera of the moribund mice which was similar to what was observed for the asymptomatic DV2-infected mice on day 5 post-infection. The amount of IL-6 in the sera of one of the moribund mice was assayed and high amount of IL-6 was detected (2690.5 pg/ml) (Table 2).

Paraplegia was observed in some of the DV2-infected mice but not for any of the mock-infected mice. As paraplegia was observed in the DV2-infected mice, hindlimbs of the mice were also harvested and quantify for DV2 titer using plaque assay (Fig 5D). Similar to what was observed for the DV2 titers of the liver, DV2 was also not detected in the limbs of every DV2-infected mouse on day 1 and day 2 post-infection and virus was detected on day 4, day 5 and day 21 post-infection. No virus was detected in the limbs of mock-infected wild-type and TLR6^*-/-*^ mice.

Paralysis of the limb could be due to presence of virus in the central nervous system [53]. In view of that, the brains of DV2-infected wild-type and TLR6^*-/-*^ mice were harvested. DV2 was able to gain entry into the brain and persisted there in both the wild-type and TLR6^*-/-*^ mice (Fig 5E). DV2 was able to replicate in the brain of TLR6^*-/-*^ mice from day 1 post-infection while DV2 was only detected in the brain of DV2-infected wild-type mice from day 3 post-infection. DV2 was only detected from day 3 post-infection in the brains of wild-type mice. DV2 can still be detected at the endpoint of the experiment in both wild-type and TLR6^*-/-*^ mice. No virus was detected in the brain of the mock-infected mice.

When the virus titers of mice which displayed lower limb paralysis were titered using plaque assay, it was found that most of the viruses were localized in the brain rather than the limb, liver or the serum (Table 2). This suggested that DV2 persisted in the brains of these mice and affected the central nervous system, leading to the paralysis. High virus titers were found in the homogenized brains of the mice which displayed symptoms of paralysis, much higher than the asymptomatic mice. One of the mice was found to have only one limb paralyzed, the virus titer in each of the limb was titered separately to determine if there would be a difference in virus titers in the two limbs. The virus titer in the paralyzed limb (1.4 x 10^3^ PFU/g) was comparable to that of the normal limb (4.0 x 10^3^ PFU/g), suggesting that paralysis was not due to virus replication in the limb (Table 2). Moreover, some of the mice which exhibited limb paralysis were not detected with DV in the limbs, further substantiating that paralysis was not due to DV in the limbs (Table 2).

**Fig 6:**
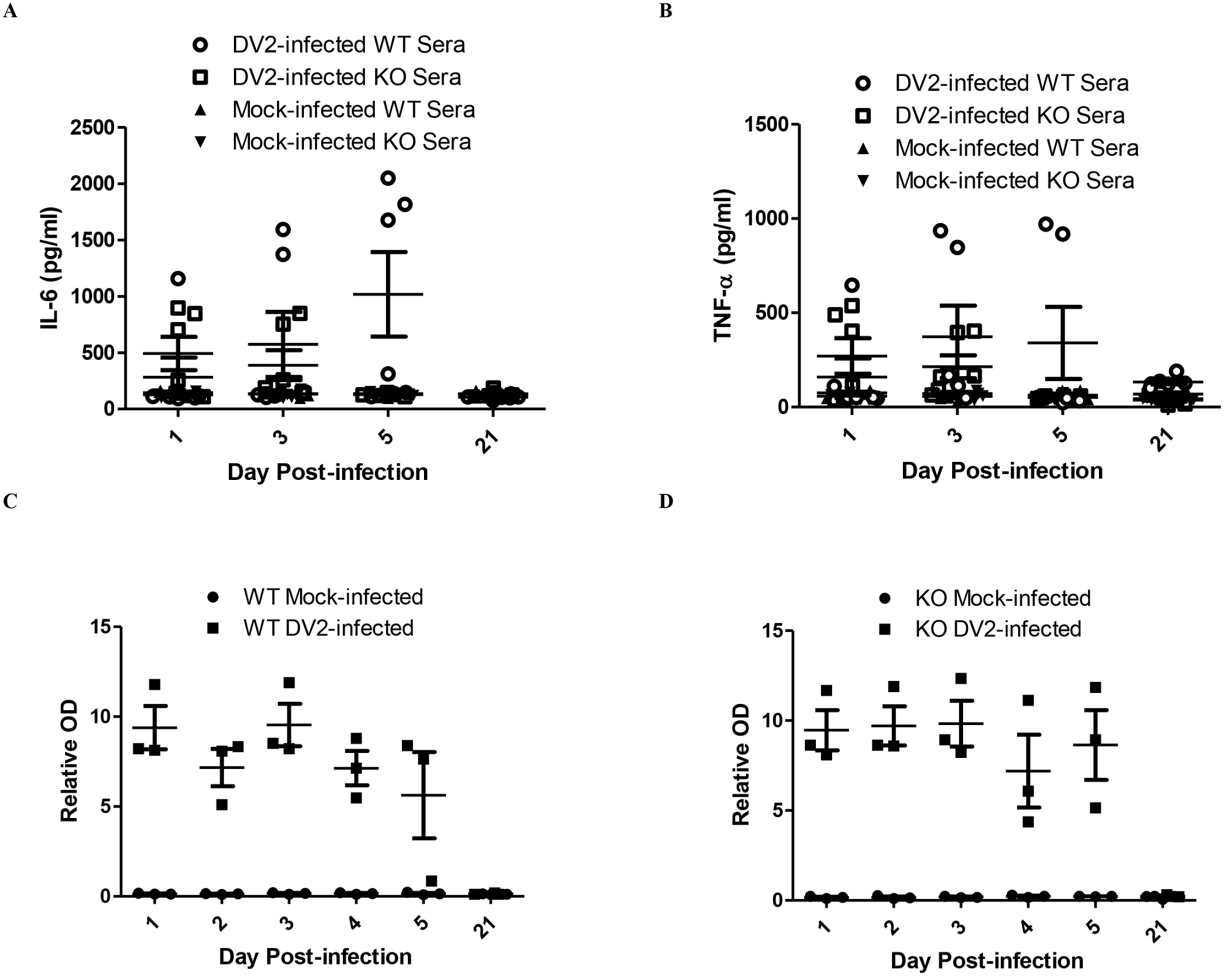
DV2-infected mice up-regulates IL-6, TNF-α and produces DV NS1 protein. ELISA was performed to quantify the amount of IL-6 (A) and TNF-α (B) in the sera of mice infected with 2.7 x 10^8^ PFU of DV2 at 1–2 day-old via intraperitoneal injection. There were 24 mice in each time point (12 DV2-infected and 12 mock-infected, 12 wild-type mice and 12 TLR6^-/-^ mice). Using Kruskal-Wallis test, the amount of IL-6 and TNF-α detected in the sera of DV2-infected wild-type and TLR6^-/-^ mice were significantly different among the respondents. Wild-type (C) and TLR6^*-/-*^ (D) mice at 1–2 day-old were infected with 2.7 x 10^8^ PFU of DV2 strain 16681. DV NS1 protein in the sera of the mice was assayed using Bio-rad Platelia Dengue NS1 Antigen detection kit.

It has been shown using the PBMC cell model that DV2 infection up-regulates IL-6 expression. IL-6 expression in sera of DV2-infected mice was investigated using ELISA. It was noticed that not all the mice up-regulated IL-6 expression upon dengue virus infection (Fig 6A). Some of the mice, both DV2-infected wild-type and TLR6^*-/-*^, remained unresponsive to the infection. The amount of IL-6 in the sera of those mice was comparable to that of the mock-infected mice. Among those that responded, DV2-infected wild-type mice secreted higher amount of IL-6 compared to that of the DV2-infected TLR6^*-/-*^ mice, indicating that TLR6 activation contributed to the IL-6 expression during dengue virus infection. This observation is similar to what was seen in the human PBMC cell model. In addition, it was noticed that IL-6 up-regulation in the sera of DV2-infected TLR6^*-/-*^ mice subsided by day 5 post-infection while that of responsive wild-type mice remained up-regulated. In general, there was an increasing trend observed in the IL-6 expression of those responsive DV2-infected wild-type mice from day 1 to day 5 post-infection.

The amount of TNF-α in the sera of DV2-infected mice was also determined using ELISA. TNF-α expression in sera of DV2-infected mice was similar to IL-6 expression (Fig 6B). Among those that responded, DV2-infected wild-type mice secreted higher amount of TNF-α compared to that of the DV2-infected TLR6^*-/-*^ mice, indicating that TLR6 activation contributed to the TNF-α expression during dengue virus infection.

DV NS1 protein was found to be the viral protein responsible for activating TLR2 and TLR6 using the PBMC model. As DV NS1 protein could be the viral protein responsible for the IL-6 and TNF-α up-regulation in the mice as well, the presence of DV NS1 protein in the sera of mice after intraperitoneal injection of DV2 was determined using Bio-Rad Platelia Dengue NS1 Antigen detection kit.

DV NS1 protein persisted in the sera of mice after injection of DV2 for both wild-type and TLR6^*-/-*^ mice (Fig 6C and 6D). In general for both wild-type and TLR6^*-/-*^ mice, the DV NS1 protein level started to decrease from day 4 post-infection and on day 5, DV NS1 protein level in one of the wild-type mice fell close to the relative OD of the mock-infected mice. The amount of DV NS1 protein in the sera of the DV2-infected wild-type and TLR6^*-/-*^ mice was comparable. This could be due to the comparable virus titers in the DV2-infected wild-type and TLR6^*-/-*^ mice, suggesting comparable replication level and thus similar DV NS1 protein production.

### 4) Knockout of TLR6 increases the survivability of DV NS1-treated mice using dengue murine model

**Fig 7:**
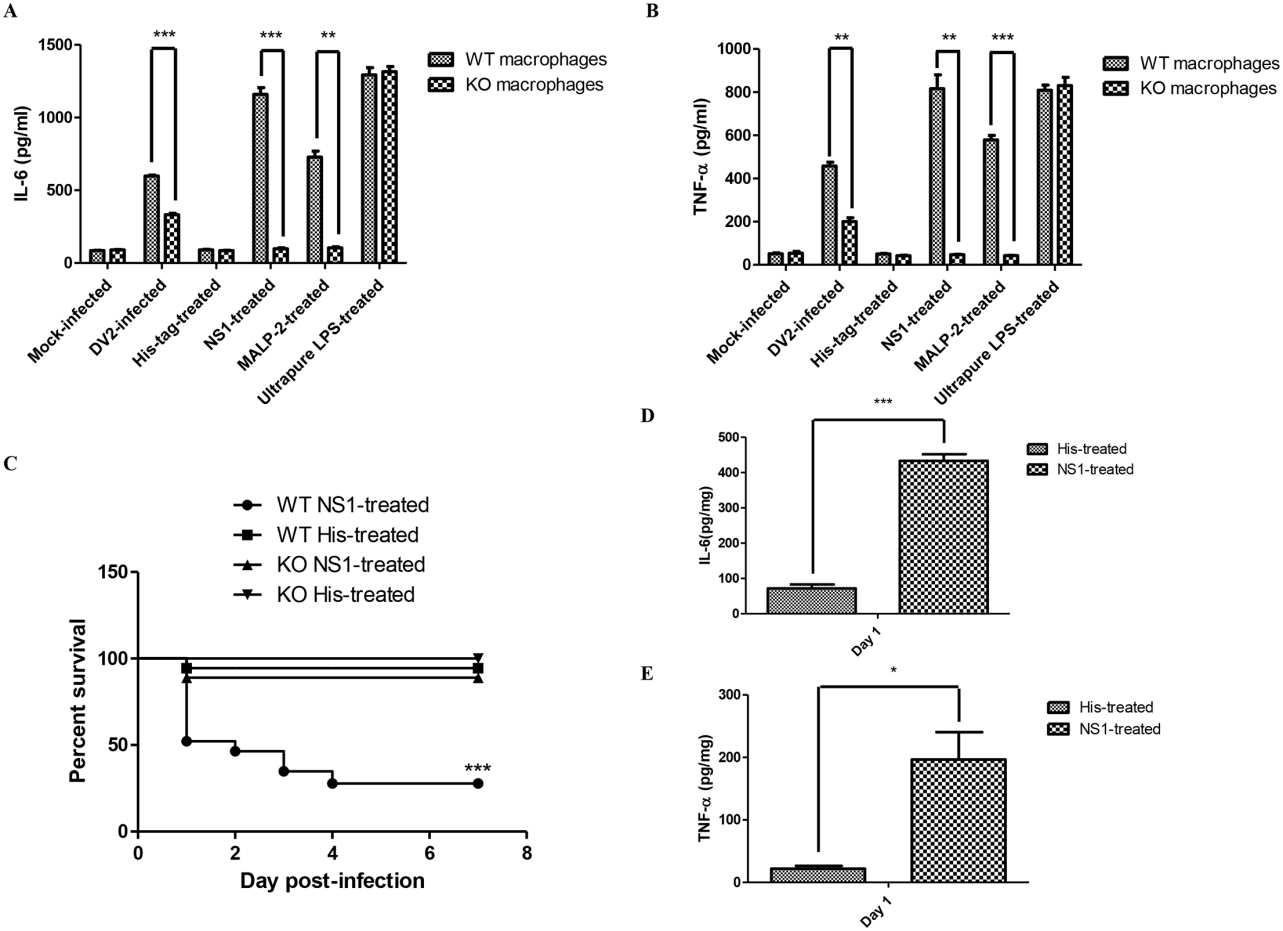
DV NS1 protein activates TLR6 of mice. ELISA was performed to quantify the amount of IL-6 (A) and TNF-α (B) secreted into the supernatant by His-tag (1 μg/ml), DV NS1 recombinant protein (1 μg/ml), MALP-2 (50 ng/ml), Ultrapure LPS (2.5 x 10^4^ EU/ml), mock-infected or DV2-infected (M.O.I of 10) wild-type or TLR6^*-/-*^ murine peritoneal macrophages on day 2 post-treatment. (C) Wild-type (23 mice) and TLR6^*-/-*^ (9) mice were injected with 20 μg of DV NS1 protein at 1–2 day-old via intraperitoneal injection. Control wild-type (18 mice) and TLR6^*-/-*^ (9) mice were injected with 20 μg of His-tag. The survival of the mice was monitored from day 1 to day 7 on a daily basis. Kaplan-Meier survival curves were shown. Log-rank test was performed to determine if there was significant difference in survivability between DV NS1-treated wild-type and TLR6^*-/-*^ mice. ELISA was performed to quantify the amount of IL-6 (D) and TNF-α (E) in the livers of wild-type mice injected with 20 μg of DV NS1 protein or His-tag at 1–2 day-old via intraperitoneal injection. Each set of samples was in triplicates.

Next, we determined if TLR6 of the mice was activated by DV NS1 protein during DV infection. The effect of DV NS1 protein on IL-6 expression of murine peritoneal macrophages was investigated. Similar to what was observed for human PBMC cell model, without the presence of TLR6, DV2-infected murine peritoneal macrophages secreted significantly lesser amount of IL-6 (Fig 7A). The amount of IL-6 produced by DV NS1 protein-treated wild-type murine peritoneal macrophages was significantly much more than DV NS1 protein-treated TLR6^*-/-*^ murine peritoneal macrophages. The level of IL-6 produced by DV NS1 protein-treated TLR6^*-/-*^ murine peritoneal macrophages was comparable to that produced by the mock-infected TLR6^*-/-*^ murine peritoneal macrophages. In the absence of TLR6, DV NS1 protein cannot stimulate the production of IL-6 by murine peritoneal macrophages. The amount of IL-6 produced by DV2-infected TLR6^*-/-*^ murine peritoneal macrophages was higher than that of the mock-infected, suggesting that the stimulation by DV NS1 protein only contributed partially to the amount of IL-6 detected in the DV2 infection. In addition, the effect of MALP-2 on the secretion of IL-6 by the murine peritoneal macrophages was eliminated in the absence of TLR6. Similar to IL-6, TNF-α production by the murine peritoneal macrophages upon stimulation with DV NS1 protein was significantly reduced in the absence of TLR6 (Fig 7B). Ultrapure LPS, a TLR4-specific agonist can induce IL-6 and TNF-α production by murine peritoneal macrophages of both wild-type and TLR6 knockout mice. These results suggested that TLR6 of mice can be activated by DV NS1 protein.

As DV NS1 protein can induce IL-6 and TNF-α production by the murine peritoneal macrophages, the effect of introducing DV NS1 protein into the mice was investigated. Wild-type and TLR6^*-/-*^ mice were injected with 20 μg of DV NS1 protein via intraperitoneal injection. The control mice were injected with 20 μg of His-tag protein. The survivability of the DV NS1 protein-treated and His-tag-treated mice were monitored for 7 days post-treatment (Fig 7C). At the endpoint, 94.4% of the His-tag-treated wild-type mice survived the treatment while only 27.8% of the DV NS1 protein-treated wild-type mice survived. At the endpoint, 100% of the His-tag-treated TLR6^*-/-*^ mice survived the treatment while 88.9% of the DV NS1 protein-treated TLR6^*-/-*^ mice survived. The survival rate of the DV NS1 protein-treated wild-type and TLR6^*-/-*^ mice were significantly different. The knockout of TLR6 increased the survivability of mice after treatment with DV NS1 protein. IL-6 (Fig 7D) and TNF-α (Fig 7E) of the treated wild-type mice were assayed. Similar to DV-infected mice, IL-6 and TNF-α in the DV NS1 protein-treated wild-type mice were found to be significantly higher than that of the His-tag-treated wild-type mice.

## Discussion

Among the PBMC, monocytes have been implicated in both the protection and immunopathogenesis of dengue [54]. Depletion of monocytes and macrophages in mice using clodronate-loaded liposomes resulted in 10-fold higher systemic DV titers, highlighting the important roles of monocytes and macrophages in DV control during an infection [55]. Ironically, monocytes were found to be the cells among PBMC that supported DV infection and the cells responsible for antibody-dependent enhancement of DV infection [56,57]. Although other cell types in the PBMC like T cells and B cells were found to be less susceptible to DV infection, they are likely to play important roles during DV infection [56]. There are evidences that cell-cell cross-talks between various immune cells in PBMC affect cytokine production during an infection [58,59]. Hence, PBMC culture would be a more representative and physiological model of infection than using monocytes alone.

In the recent years, it has become evident that PRRs play a major role in infectious and even in non-infectious diseases [60,61]. One family of PRRs, the TLR family has emerged as a key component of the innate immune system and it can activate signals which are crucial for the initiation of adaptive immune responses [61]. Studies in recent years have shown the presence of mRNA and protein expression of TLRs in various immune and non-immune cells [62–64]. In our study, TLR2 and TLR6 were found to be up-regulated in PBMC upon DV infection. This up-regulation suggested the possible involvement of TLR2 and TLR6 in dengue virus infection.

TLR6 was found to be up-regulated by PBMC on day 3 post-infection (Fig 1C). As TLR2 is partner of TLR6, its expression by PBMC during dengue virus infection was also investigated. Like what was observed for TLR6, TLR2 was found to be up-regulated by PBMC on day 3 post-infection (Fig 1D). The mechanism of TLR2 regulation has not been fully elucidated [65–68]. It was reported that chromatin remodelling involving DNase I and restriction enzyme occurs at TLR2 promoter region following infection [67]. This remodelling of chromatin increases accessibility of transcription factors resulting in greater transcription of TLR2 [67]. In addition, two pathways were found to be important for TLR2 regulation. IKKβ-IκBα-dependent NFκB pathway activation and MKK3/6-p38α/β pathway inhibition are essential for TLR2 expression [65]. One study supported the involvement of NFκB in TLR2 expression. Pyrrolidine dithiocarbamate (PDTC), a pharmacologic inhibitor of NFκB was shown to prevent the up-regulation of TLR2 by TLR2 and TLR4 agonist [66]. On the other hand, the up-regulation of TLR6 is not well-studied and remained unclear.

Upon dengue virus infection, PBMC secretes both IL-6 and TNF-α (Fig 2A and 2B). This is consistent with what was observed in the sera of dengue patients. Dengue patients’ sera have been found to contain high level of IL-6 [20,69,70]. Similarly, TNF-α level was also increased in the sera of dengue patients [20,69,70]. Similar to monocytes, IL-6 and TNF-α are implicated in both the protection and immunopathogenesis of dengue virus infection [71,72]. Upon sensing the presence of foreign microbes through recognition of PAMPs, biological mediators like IL-6 and TNF-α are released. Although these mediators initiate and regulate the inflammatory response and adaptive immune response to eliminate foreign microbes, they have also been found to be involved in lethal manifestations like septic shock syndrome, vascular leakage and cachexia, resulting from disease, infection or injury [73–75]. This provided evidence that the manifestation of illness could also be caused by the host own immune system, not necessarily by exogenous pathogens.

It was noticed that IL-6 production by the DV2-infected PBMC (Fig 2A) was lower than that of the antibody-treated DV2-infected PBMC (Fig 2C). This difference could be due to the presence of antibodies which are originated from rabbit and mouse. The presence of foreign proteins can trigger immune response. Another possibility contributing to the difference in IL-6 production observed could be donor variability. The PBMC used for the two set of experiments were from different donors.

The dengue viral protein responsible for the activation of TLR6 and TLR2 were first screened using ELISA. Among the 10 dengue viral proteins, only DV NS1 protein up-regulated both IL-6 and TNF-α expression of PBMC (Fig 3A and 3B), making it the most likely candidate. DV NS1 protein was documented to be secreted by infected cells and the presence of DV NS1 protein was detected in the sera of patients [76–78]. Moreover, the amount of DV NS1 protein in the sera of patients was found to correlate with the severity of the dengue disease [76]. This correlation suggested that DV NS1 protein plays an important role in the pathogenesis of dengue disease. In addition, DV replication was found to be critical for the up-regulation of IL-6 and TNF-α during DV infection as UV-inactivated DV did not induce the up-regulation. DV NS1 protein being a non-structural protein requires DV replication to be synthesized. Hence, DV NS1 protein being the dengue viral protein fits the results we obtained using the PBMC *in vitro* cell model.

In this study, we have developed a murine model for dengue virus infection using 1–2 day old C57BL/6 mouse. Although consistent viremia was detected in the mice infected at 1–2 day old for both wild-type and TLR6^*-/-*^ mice, the virus titers were observed to decrease as the day of infection progressed (Fig 5B). This suggested that the DV2-infected mice were able to mount an effective immune response to fight the infection. The fast clearance of DV may suggest that the innate immunity is sufficient for the clearing. Published studies have demonstrated that innate immunity was sufficient to clear DV infection using human cell-engrafted *scid* mice [79,80]. Similar trend was observed for the virus titers in the livers of the DV2-infected mice for both wild-type and TLR6^*-/-*^ mice. However, the virus titers of the livers were not as consistent compared to that of the sera, only a few of the livers of DV2-infected mice were detected to contain infectious DV2 (Fig 5C). A point to be taken into consideration is that the DV detected in the liver could be contributed partially by the virus found in the blood. However, the blood contamination has been minimized as the blood of the pups was harvested before the harvest of the liver. Hence, the liver should contain minimal amount of blood when harvested. The observation that virus was detected in all the serum samples but not in all the liver samples of infected pups on day 1 and day 2 post-infection showed that the above mentioned contamination was kept to a minimum.

One clinical sign observed in dengue patients and the murine model is paralysis of extremities [52]. The occurrence of paralysis was observed to be between day 10 to day 14 of infection (Table 2). Paralegia was not unique to the murine model used in our studies. Paralegia was also observed in other murine models of DV infection and the time of development was similar [81,82]. AG129 mice were reported to develop paralysis within 7 to 14 days post-infection [82]. The development of paralysis was faster than the wild-type counterpart of the mice and thus the authors attributed the difference to the AG129’s deficiency in IFN receptors.

Among the asymptomatic mice, virus was detected in the brain as early as day 1 post-infection for the DV2-infected TLR6^*-/-*^ mice and the virus persisted in the brains of the mice till the endpoint of the experiment. This suggested that virus clearance is least efficient in the brains of the mice. Similar trend was observed for AG129 murine model [82]. AG129 mice harboured DV in the extraneural tissues and neural tissues on day 3 post-infection, with higher viral titers in the extraneutral sites than the neural sites. By day 7 post-infection, virus was only detected in the neural tissues. This result supported our data (Fig 5B–5E). It was noticed that the viral loads in the brain of the asymptomatic mice (< 10^4^ PFU/g) were much lower than that of the mice displaying hindlimb paralysis (2.8 x 10^4^ PFU/g, 5.2 x 10^5^ PFU/g, 8.2 x 10^5^ PFU/g, 7.1 x 10^5^ PFU/g) (Table 2). Hence, extensive replication of DV in the brain of the mice could have resulted in the paralysis observed in the mice. Similarly, AG129 mice with paralysis were reported to carry high viral loads in the brain [82]. The viral loads reported were comparable to ours, between 10^4^ to 10^6^ PFU/g (Table 2).

Mice which were not sacrificed but were monitored for disease progression, recovered two days after the onset of paralysis. The brain, liver and limb of one of the mice were harvested and quantified for virus. No virus was detected in the liver and limbs of the recovered mouse while virus was still detected in the brain. The viral load (2.12 x 10^4^ PFU/g) was still higher than that of the asymptomatic mice but lower than that of the symptomatic mice (Table 2 and Fig 5E). This suggested that viral load in the brains of the mice can be controlled by the mouse immune system even though the clearing of the virus in the brain was not as efficient as compared to the sera, livers and limbs. The microglial cells are the main cell type of the innate immune system in the brain [83]. The microgial cells also express TLRs and produce pro-inflammatory mediators in response to TLR ligands [84,85]. Human microgial cells express high levels of TLR2 and TLR3, moderate levels of TLR4, TLR5, TLR6, TLR7 and TLR8 but low level of TLR9 [86]. Mouse microgial cells express similar TLRs except for TLR5 [87]. As there is no lymphatic system in the brain for immune cells to migrate through and microglial cells are poor antigen-presenting cells, the immune responses in the brain are limited [83]. This may explain why the virus can persist in the brain for a longer time in comparison to other organs and sera.

As human IL-6 and TNF-α were detected in our human cell-based model upon DV infection, murine IL-6 and TNF-α were assayed for in the sera of the DV2-infected mice. Unlike what was observed for the cell-based model, IL-6 and TNF-α were only detected to be up-regulated in the sera of some of the DV2-infected mice (Fig 6A and 6B). For both wild-type and TLR6^*-/-*^ mice, the level of IL-6 and TNF-α detected in some of the DV2-infected mice were comparable to that of the mock-infected mice. This suggested only some of the DV2-infected mice responded to the DV-infection by up-regulation of IL-6 and TNF-α. This high variability of hyporesponsiveness of young mice to stimulation was also documented by other research groups [88–90]. Among the responsive mice, the IL-6 and TNF-α of the DV2-infected wild-type mice were higher than the DV2-infected TLR6^*-/-*^ mice and the up-regulation lasted for a longer time. Knockout of TLR6 reduced IL-6 and TNF-α production, suggesting TLR6 activation contributed to IL-6 and TNF-α production in mice during DV infection.

As DV NS1 protein was found to be the viral protein that is activating TLR6, the duration in which DV NS1 protein remained in circulation in the mice injected with DV was investigated. The presence of DV NS1 protein was detected in all the sera of DV2-infected wild-type and TLR6^*-/-*^ mice using Bio-rad platelia kit DV NS1 antigen detection kit from day 1 to day 5 (Fig 6C and 6D). The level of DV NS1 protein detected from the DV2-infected wild-type mice was not significantly different from that of the DV2-infected TLR6^*-/-*^ mice. This was probably a consequence of similar virus titers in the sera of the DV2-infected wild-type and TLR6^*-/-*^ mice. DV NS1 protein remained in circulation in the mice longer than DV (Figs 5B, 6C and 6D). DV NS1 protein remained detectable in the sera of DV2-infected mice on day 5 post-infection while DV were no longer detected in most of the sera by day 4 post-infection. Slower rate of DV NS1 protein clearance compared with DV from the plasma of dengue patients were also reported [76]. DV NS1 protein can be detected for a longer period of time in the sera of dengue patients compared to DV [91].

The presence of DV NS1 protein level in the mice contributes to IL-6 and TNF-α level in the mice. DV NS1 protein level remained relatively high from day 1 to day 5 post-infection. This could be the reason why wild-type mice still observed high IL-6 and TNF-α expression even when virus titer dropped to 0 PFU/ml in the sera for most of the mice while IL-6 and TNF-α of TLR6^*-/-*^ mice dropped after the elimination of DV from the sera (Fig 6A and 6B). This may suggest that IL-6 and TNF-α in the sera at the later part of infection was mostly contributed by DV NS1 protein activating TLR6.

The Kaplan-meier survival plot was used to estimate the survival of the wild-type and TLR6^*-/-*^ mice population after DV infection for over 21 days. The survival plots of the DV2-infected wild-type and TLR6^*-/-*^ mice intercept, indicating that the probability of survival for one population of the mice were higher for a period of time during DV infection but became lower compared to the other population as the infection progresses (Fig 5A). The DV2-infected TLR6^*-/-*^ mice have a lower survival probability at earlier time points and the DV2-infected wild-type mice have a lower survival probability at later time points. This could be due to the replication of DV in the brain of the TLR6^*-/-*^ mice. Viral loads were detected in the brain of the TLR6^*-/-*^ mice on day 1 post-infection but not for wild-type mice. This suggested that the brains of pups were more vulnerable to DV infection in the absence of TLR6. Sensing of DV through other PRRs may be more limited for the microgial cells during the early development of the mice and thus TLR6 appeared to play a more critical role. This vulnerability decreased with age as TLR6^*-/-*^ mice suffering from paraplegia were found to be able to recover from it. The overall survival probability of wild-type mice during DV2 infection was lower than TLR6^*-/-*^ mice. In the absence of TLR6 activation, the overall survival probability of the mice to DV infection increased. This suggested the involvement of TLR6 in the immunopathogenesis of DV infection. Activation of TLR2 and TLR6 by DV NS1 protein up-regulates IL-6 and TNF-α. High expression of IL-6 and TNF-α have been shown to be associated with fatality of mice [92,93]. Prolonged up-regulation of IL-6 and TNF-α due to stimulation of TLR6 by DV NS1 protein may be the cause of death for the wild-type mice. Prolonged up-regulation of IL-6 and TNF-α may increase the risk of the mice developing complications from the proinflammatory cytokines.

Murine peritoneal macrophages from 4-week old wild-type and TLR6^*-/-*^ C57BL/6 mice were used to further verify the involvement of TLR6 in IL-6 and TNF-α expression during DV infection. Murine peritoneal macrophages were widely used to elucidate TLR ligands and TLR6 ligands were among those tested [32,33,94,95]. MALP-2 was also used to further confirm the functionality of TLR6 of the wild-type and TLR6^*-/-*^ murine peritoneal macrophages. Wild-type murine peritoneal macrophages up-regulated both IL-6 and TNF-α upon stimulation by MALP-2 while TLR6^*-/-*^ murine peritoneal macrophages were non-responsive. During DV infection, TLR6^*-/-*^ murine peritoneal macrophages produced significantly less IL-6 and TNF-α compared to that of the wild-type murine peritoneal macrophages. This corroborates the result obtained from the sera of the mice. Knockout of TLR6 did not completely abrogate IL-6 and TNF-α up-regulation during DV infection, suggesting TLR6 activation only partially contributed to the IL-6 and TNF-α detected during DV infection. The redundancy of pathogen sensing pathways was documented [96]. One pathogen can be recognized by multiple PRRs and the signalling pathways activated downstream of TLRs have redundancy [97]. The synergistic effect of activating more than one PRR has also been reported. Synergy between TLR2 and TLR4 can potentiate the up-regulation of cytokine production [98]. This observation may also provide some explanation on why wild-type and TLR6^*-/-*^ mice did not have significant difference in virus detected in the sera of the mice. Knockout of TLR6 did not prevent the activation of macrophages. The macrophages can still sense the presence of pathogen through other PRRs and gets activated to produce IL-6 and TNF-α. Similar to our human PBMC model, DV NS1 protein stimulated the production of IL-6 and TNF-α by wild-type murine peritoneal macrophages. TLR6^*-/-*^ murine peritoneal macrophages were unresponsive to DV NS1 protein stimulation, suggesting DV NS1 protein activates TLR6 of murine peritoneal macrophages to produce IL-6 and TNF-α. Using TLR6^-/-^ murine cellular model, TLR2 and 6 antibody blocking assay and SEAP reporter assay, DV NS1 protein has been shown to be the viral protein responsible for TLR2/6 stimulation during DV infection and both receptors are required. However, whether the stimulation is direct or indirect has not been elucidated. Studies have demonstrated that host-derived molecules may also stimulate TLR signalling [99]. Hence, there is a possibility that DV NS1 protein may stimulate the release of endogenous ligands to trigger TLR2 and TLR6 activation rather than binding to TLR2/6 complex itself

It was found that mice injected with DV NS1 protein alone without DV can induce up-regulation of IL-6 and TNF-α (Fig 7D and 7E). Hence, the result suggested that DV NS1 protein contributed to the up-regulation of IL-6 and TNF-α production observed in DV2-infected wild-type mice (Fig 6A and 6B). In addition, results from the murine peritoneal macrophages suggested that DV NS1 protein stimulates IL-6 and TNF-α production primarily through TLR6. Together, these results suggest that the higher survivability of the TLR6^-/-^ mice during DV infection could be due to their non-responsiveness to DV NS1 protein. The survival rate of the DV NS1 protein-treated wild-type mice (27.8%) was lower than that of the DV-infected wild-type mice (61.4%). This could be due to the amount of DV NS1 protein injected was more than what was produced in the mice by the DV infection.

It was shown in our studies that DV NS1 protein is able to activate TLR2 and TLR6 to induce up-regulation of IL-6 and TNF-α. This production of cytokines could be the cause of the development of dengue hemorrhagic fever as cytokines were found to play important roles in several viral hemorrhagic fevers [100,101]. Furthermore, cytokines were found to have prognostic value in DV infection in other studies [20,102,103]. All these findings suggest that a possible treatment for dengue would be to control the proinflammatory cytokine production during DV infection. It was reported that when an immunomodulator, tetracycline hydrochloride was administered into Tick-Borne Encephalitis virus patients, the concentration of IL-6 and TNF-α were reduced and the patients have a faster clinical recovery [101]. This study suggested that modulation of the amount of IL-6 and TNF-α can have a positive effect on patients with viral hemorrhagic fevers. Since TLR6 activation during DV infection can contribute to the production of proinflammatory cytokines, immunomodulation approaches that target TLR6 can reduce the proinflammatory cytokines. The reduction of proinflammatory cytokines can potentially prevent the progression of the disease to the more severe forms.

Recent studies have shown that TLRs may be responsible for the manifestation of autoimmune diseases, allergy, cancer, infectious diseases and sepsis [104,105]. In our study, the activation of TLR6 decreases the survival of mice during DV infection, suggesting a role for TLR6 in the immunopathogenesis of DV infection. The roles of TLRs in human diseases are not fully understood but there are *in vitro* and animal model data to support TLR roles in disease initiation and progression [97,106]. There is a growing interest in exploring TLRs as the therapeutic targets for these diseases [97,104–107]. It was proposed that inhibition of TLR function might limit disease pathogenesis in conditions such as sepsis, rheumatoid arthritis and systemic lupus erythematosus, in which the immune system is inappropriately overactive [97,104,106]. Antimalarial drugs such as hydroxychloroquine which act as a TLR7, TLR8 and TLR9 antagonist are used for the treatments of rheumatoid arthritis and systemic lupus erythematosus [106,108]. TLR2 has been implicated in the pathogenesis of systemic lupus erythematosus, diabetes, Alzheimer’s disease [109,110]. A TLR2-specific monoclonal antibody, OPN-305 which inhibits TLR2-mediated proinflammatory cytokine production is being tested for the potential treatment of inflammatory diseases [106]. Drugs or antibodies that target TLR2 are likely to have an effect on TLR2 and TLR6 signaling as shown by PBMC model, in which inhibition of IL-6 and TNF-α was achieved by the blocking of either TLR2 or TLR6. The host may not be vulnerable to pathogens in the duration of TLR6-targeted therapy, due to the redundancy of PRR pathways. TLR6-targeted therapies have a potential for intervention in dengue virus infection and amelioration of disease symptoms. Besides using small-molecule agonists or antagonists for targeting TLRs, the use of microRNAs in the regulation of TLRs may be available in the near future [111,112].

In our study, we have found that TLR2 and TLR6 were involved in the detection of the presence of DV during DV infection. However, mice without TLR6 were still able to secrete IL-6 and TNF-α during DV infection. Therefore, other PRRs are also likely to be involved. It would provide a better understanding of the DV infection if the identities of those PRRs were elucidated. Some of the proposed PRRs are TLR3, TLR7 and TLR8 [28].

In conclusion, DV NS1 protein is found to be responsible for triggering TLR2 and TLR6 during DV infection in our study. This stimulation partially contributes to IL-6 and TNF-α expression during DV infection. Activation of TLR6 may play a role in the immunopathogenesis of DV infection in the mice as survivability of the mice increased in the absence of TLR6. Lastly, our results provide an insight into the possibility of using TLR6 antagonist in therapeutic treatment for DV infection.

## Materials and Methods

### Ethics statement

Human peripheral blood mononuclear cells (PBMC) were isolated with informed consent from healthy blood donors as whole blood donation, from the Division of Haematology, Department of Laboratory Medicine, National University Hospital, Singapore and was approved by National University of Singapore Institutional Review Board (NUS-IRB: 10-072E). Animal research was approved by NUS IACUC (protocol no: 090/10, R15-0033, BR023/10, BR14-1255). The mice were anesthesized using isoflurane. Euthanasia was performed using carbon dioxide asphyxiation, followed by cervical dislocation.

### Cell culture

The Baby Hamster Kidney (BHK) cells (ATCC), Human Embryonic Kidney (HEK) 293 cells and *Aedes albopictus* C6/36 cells were grown in RPMI-1640 (Sigma Aldrich) supplemented with 10% fetal calf serum [(FCS), PAA], DMEM (Sigma Aldrich) supplemented with 10% FCS and L-15 media supplemented with 10% FCS respectively. Human peripheral blood mononuclear cells (PBMC) were isolated with informed consent from healthy blood donors as whole blood donation, from the Division of Haematology, Department of Laboratory Medicine, National University Hospital, Singapore and was approved by National University of Singapore Institutional Review Board (NUS-IRB: 10-072E). PBMC were isolated from the donors’ buffy coats by centrifugation on a density gradient (400x g/30 mins in Ficoll-Paque Plus, GE Health Science) according to manufacturer’s procedures. Isolated PBMC were grown in RPMI-1640 supplemented with 10% FCS and 1% penicillin-streptomycin of concentration: 10, 000 units penicillin and 10 mg streptomycin/ml. LPS-treated PBMC were added lipopolysaccharide (Sigma Aldrich, L-2630) into PBMC culture medium. Ultrapure LPS-treated PBMC were added ultrapure lipopolysaccharide (InvivoGen, LPS-EB Ultrapure). His-tag-treated PBMC were added 1 μg/ml of His-tag (Abcam ab14943) into PBMC culture medium. DV NS1 protein-treated PBMC were added 1 μg/ml of DV NS1 protein (Abcam ab64456) into PBMC culture medium.

### Virus strain and infection

Dengue virus serotype 2 (DV2), strain (Den2STp7c6), a low passage isolate from a dengue-infected patient in Singapore and DV2 strain 16681, a kind gift from Professor Gubler from DUKE NUS were used in this study. The virus was propagated in C6/36 cells. PBMC were transferred separately into 50 ml falcon tubes and centrifuged at 300x g for 5 mins to remove the culture medium. PBMC were infected with DV2 at a multiplicity of infection (MOI) of 10 and incubated at 37°C for 1.5 hour with intermittent shaking. The cells were washed with PBS once to remove residual virus before RPMI-1640 medium with 10% FCS was added to the cells. PBMC were seeded into each well of 24-well plates (NUNC). HEK 293 cells were seeded in each well of 24-well plates 1 day before infection. Prior to infection, the culture medium was aspirated from the wells and the HEK 293 cells were infected with DV2 at an MOI of 10 with incubation at 37°C for 1.5 hour with intermittent shaking. The cells were subsequently washed with PBS before culture medium was added to the cells. Supernatant from uninfected C6/36 culture was denoted as the mock-infected controls. RPMI-1640 supplemented with 10% FCS and 1% penicillin-streptomycin was the culture medium used for PBMC while DMEM supplemented with 2% FCS was used for HEK 293 cells. UV-inactivated virus was obtained by irradiation of the virus under the ultraviolet lamp for 1.5 hours. The UV-inactivated virus in the medium was then purified in 100 kDa nominal molecular weight limit centricons (Millipore, UFC910096) and centrifuged at 4000x g for 25 mins in a swing-out centrifuge. PBS was then added into the centricons to wash the virus and centrifuged again at 4000x g for 25 mins. The virus was then collected and reconstituted with L-15 medium. The inactivation of the virus by UV-irradiation was confirmed by performing virus plaque assay.

### Virus plaque assay

Plaque assay was carried out to quantify the number of infectious virus particles using BHK cells. Briefly, BHK cells were cultured to approximately 80% confluency in 24-well plates. The virus stock was 10-fold serially diluted from 10^−1^ to 10^−6^ dilution in RPMI 1640. BHK monolayers were infected with 100 μl of each virus dilution. After incubation in 5% CO_2_ atmosphere at 37°C for 1 hour with rocking at 15 mins intervals, the medium was aspirated and 1 ml of 1% (w/v) carboxymethyl cellulose in RPMI supplemented with 2% FCS was added to each well. After 6 days of incubation at 37°C in 5% CO_2_ incubator, the cells were fixed and stained for 1 hour with 200 μl of 1% crystal violet in staining solution. After thorough rinsing with water, the plates were dried and the virus plaques were scored visually.

### Western blot

Cell pellets were lysed using CelLytic M cell lysis reagent (Sigma Aldrich) with EDTA-free protease inhibitor cocktail (Roche) for 10 mins on ice. The total cellular protein in samples was quantified by Bradford Assay (Bio-Rad). 15 μg of protein was loaded in each lane and separated by SDS-PAGE before being transferred onto a nitrocellulose membrane via the semi-dry transfer system (Bio-Rad). Western blot was performed to detect human TLR6, using rabbit IgG anti-TLR6 (sc-30001, Santa Cruz Biotechnology) (1:200 dilution) and TLR2, using rabbit IgG anti-TLR2 (ab86754, Abcam) (1:200 dilution). Blots were incubated with HRP-conjugated goat anti-rabbit IgG (H+L) secondary antibody (Pierce) (1:2500 dilution). Analyses were performed using enhanced chemiluminescence detection system with Pierce ECL Western Blotting Substrate. The density of the bands was quantified using GelQuant.NET software provided by biochemlabsolutions.com.

### Flow cytometry

PBMC infected with DV2 at an MOI of 10 or mock-infected were transferred from 24-well plates into 15ml falcon tubes. The cells in the falcon tubes were centrifuged at 300x g for 5 mins. The supernatant were then discarded and 5ml of PBS were added for the washing of the cells. The cells were spun down once more to remove the PBS. PBS containing 5% BSA was used to resuspend the cells before the cells were incubated on ice for 20 mins. Primary antibody [anti-TLR6 (Santa Cruz SC-30001), anti-TLR2 (Santa Cruz SC-21759) or anti-CD14 (Millipore CBL453F)] was then added at a dilution of 1: 200 to the cell suspension and incubated on ice for 30 mins. The cells were then spun down and the supernatant removed. 5 ml of PBS was used to wash the cells before the cells were spun down again to remove the PBS. Following that DyLight 633/FITC-conjugated goat anti-rabbit or anti-mouse IgG (H+L) secondary antibody (Pierce), was added at 1: 200 and incubated on ice for 30 mins. The cells were then washed twice with 5ml of PBS before fixing using 4% paraformaldehyde at room temperature for 10 mins. After which the cells were washed and resuspended in 1 ml of PBS. For the staining of two different antigens in the same sample, the above procedure of staining was repeated once more using primary antibodies derived from a different species. The cells were analyzed using Beckman Coulter CyAn ADP Analyzer. Samples were gated to exclude cell debris.

### Immunofluorescence staining

PBMC were stained with anti-TLR6 antibody (Santa Cruz, SC-30001) or anti-TLR2 (Santa Cruz SC-21759) and anti-CD14 (Millipore CBL453F) at a dilution of 1:200 for 30 mins, followed by FITC-conjugated goat anti-rabbit IgG (H+L) secondary antibody for 30 mins and fixed using 4% paraformaldehyde. The cells were then incubated with 4’-6-Diamidino-2-phenylindole (DAPI) at a concentration of 300nM for 5 mins at room temperature. The cells were spun down at 300x g for 5 mins and washed twice in 5 ml of PBS. The cells were resuspended in 10 μl of PBS. 10 μl of the cell suspension was placed onto a glass slide, mounted on coverslip and viewed under the microscope (IX81 Olympus, Japan) at 1000x magnification.

### Quantification of cytokines using enzyme-linked immunosorbent assay

Quantification of cytokines (IL-6 and TNF-α) was carried out using sandwich enzyme-linked immunosorbent assay (ELISA) which was performed in 96-well plate. ELISA for human and murine IL-6 and TNF-α were performed using commercial kits (BD biosciences, Pharmingen) and according to manufacturer’s protocol. Briefly, 100 μl of anti-IL-6 or anti-TNF-α antibody diluted 1:250 with coating buffer were added into each well to coat the antibody onto the plate through an overnight incubation at 4°C. The plates were then washed three times using wash buffer (PBS with 0.05% Tween-20) before blocking using 200 μl of assay diluent per well. After adding the standards and the samples, the plates were washed three times using wash buffer and incubated with 100 μl of anti-IL-6 or anti-TNF-α biotinylated antibody and streptavidin-conjugated horseradish peroxidase diluted 1:250 with assay diluent for an hour. The plate was then washed seven times, followed by adding tetramethyl benzidine substrate solution to each well. Absorbance was measured using ELISA reader (Tecan) at wavelength of 450 nm with reference wavelength of 570 nm. The concentrations of the cytokine in experimental samples were determined from a standard curve with known concentrations of the cytokine. Samples were performed in triplicates.

### Blocking of TLR2 and TLR6 using neutralizing antibodies

PBMC were incubated with TLR2 or TLR6 blocking antibodies (IgG1) (InvivoGen, San Diego, USA) at a concentration of 1000 ng/ml for 30 mins on ice. Unbound antibodies were washed off with PBS before infection or mock-infection was performed. For PBMC to be blocked by both TLR2 and TLR6 blocking antibodies, 500 ng/ml of each antibody were used instead. Normal mouse IgG1 (Santa Cruz Biotechnologies, Santa Cruz, USA) from unstimulated mice was used as isotype control at a concentration of 1000 ng/ml.

### Detection of NFκB activity using secreted alkaline phosphatase reporter assay

Activation of NFκB was determined using a reporter plasmid (pNF-κB/SEAP, IMGENEX) which expresses secreted alkaline phosphatase (SEAP) protein under the control of the NFκB promoter. These plasmids were transfected into HEK 293 cells using Invitrogen Lipofectamine LTX according to manufacturer’s protocol. SEAP catalyzes the hydrolysis of p-Nitrophenyl phosphate producing a yellow product that can be read using ELISA reader at 405 nm. Stable cell clones of the transfected cells were obtained by selection using G418 (PAA) at 500μg/ml. In brief, 2 x 10^5^ transfected HEK 293 cells were seeded into 24-well plate 1 day prior to treatment. The cells were then treated under various conditions [infected with DV2 at an MOI of 10, mock-infected, DV NS1 recombinant protein (1 μg/ml), LPS (5 μg/ml) or MALP-2 (50 ng/ml) (Imgenex, IMG-2206) added into the culture medium]. Supernatant were harvested 1 to 3 day post-treatment. The amount of SEAP in the supernatant was assayed according to manufacturer’s protocol and read using an ELISA reader (Tecan) at wavelength of 405 nm.

### Expression of TLR2 and TLR6 in HEK 293 cells

TLR2 and TLR6 in HEK 293 were expressed using a plasmid co-expressing the human TLR2 and TLR6 genes (pDUO-hTLR6/TLR2, InvivoGen) which was transfected into the NFκB-SEAP HEK 293 cell clones obtained as mentioned in the previous paragraph. Stable cell clones of the transfected cells were obtained by selection using both blasticidin (Invitrogen) at 10 μg/ml and G418 (PAA) at 500 μg/ml. The expression was confirmed with immunoblotting after the transfected cells were stained for TLR2 or TLR6 using the antibodies mentioned above.

### Production and purification of dengue viral recombinant proteins

Dengue viral recombinant proteins (Capsid, PrM, envelope, NS1, NS2A, NS2B, NS3, NS4A, NS4B, NS5) with 6x his-tag and protein tag (6x his-tag) at concentration of 1 mg/ml were expressed using BaculoDirect Baculovirus Expression System (Invitrogen) according to manufacturer’s protocols.

### Detection of DV NS1 antigen

Sera of mock-infected and DV2-infected mice were harvested for DV NS1 antigen detection assay using the Platelia Dengue NS1 Antigen detection kit (Bio-Rad, #72830) for day 1 to day 5 and day 21 post-infection. The relative amount of DV NS1 protein was measured in optical density and was read using Tecan plate reader at wavelength of 450 nm with reference wavelength of 620 nm.

### Strains of mice

C57BL/6 mice used in this study were obtained from InVivos and the former NUS CARE (NUS, Singapore). TLR6 knock-out C57BL/6 breeder mice were obtained from Oriental BioService, Kyoto, Japan and bred in NUS, Singapore under NUS IACUC approved breeding protocols, BR023/10 and BR14-1255. The use of mice for this study was approved by NUS IACUC under protocols, 090/10 and R15-0033.

### DV NS1 protein treatment, His-tag treatment, infection and mock-infection of mice

One to two days old C57BL/6 mice were infected with 5.4 x 10^8^ PFU/ml of 16681 DV2 via intraperitoneal injection (IP) at a volume of 0.05 ml/g. For mock-infection, C6/36 culture supernatant of the same volume as the virus was injected instead. For DV NS1 protein treatment, one to two days old C57BL/6 mice were injected with 20 μl of DV NS1 recombinant protein of concentration 1 mg/ml. For His-tag treatment, 20 μl of His-tag of concentration 1 mg/ml was injected instead.

### Harvesting blood, peritoneal fluid and tissues of mice

Mice were euthanized before blood was collected by cardiac puncture. The blood was left to clot at room temperature and centrifuged at 3300x g for 5 mins to obtain the serum. It was observed that some mice had a bulge at the site of injection on day 1 post-infection. Peritoneal fluid was extracted from the bulge using 27 G needle and syringe for virus quantification as well. Brains of mice were harvested by removing the skin on top of the head and making an incision at the centre of the scalp using scissors. Livers of mice were harvested by making an incision at the abdomen. Hindlimbs of mice were harvested by cutting the hind limbs of the mice at the pelvis joint. Brains, livers or hind limbs of mice were placed in hard tissue homogenizing tube containing ceramic beads (Precellys, Bertin, Germany). The weight of the tissues in each of the tubes was recorded and 0.5 ml of PBS was added to each tubes. The tissues in the tubes were homogenized using a tissue homogenizer (Precellys, Bertin, Germany). The conditions used were 6500 rpm for 10 secs with 3 repetitions and 5 secs rest in between. The tubes were then centrifuged at 3500x g for 10 mins. The supernatant was collected in a new eppendorf tube and centrifuged at 10,000x g for 10 mins. DV2 in the serum or peritoneal fluid or supernatant of homogenized tissues were determined using plaque assays. 1% penicillin-streptomycin, 1% amphotericin B (MP Biomedicals, Southern California, USA) and 0.5% gentamycin (PAA, GE Healthcare, Piscataway, USA) were added to the overlay medium. As the volume of the serum or peritoneal fluid harvested from each of the pups may be less than 100 μl, there may not be neat sample and the calculation of PFU/g was adjusted according to the volume of sample used. IL-6 and TNF-α in the supernatant of homogenized tissues were determined using ELISA.

### Stimulating production and harvesting of murine peritoneal macrophages

4% thioglycollate medium was prepared and autoclaved. 4-week old mice were injected with 1 ml of 4% thioglycollate medium via intraperitoneal injection. Four days after injection, the mice were euthanized and the skin around the abdomen of the mice was removed to expose the intraperitoneal cavity. Ice cold PBS was injected into the intraperitoneal cavity without bursting the peritoneal membrane. Precaution was taken to avoid puncturing any organ or intestine. The abdomen of the mice was gently massaged before withdrawing the PBS containing macrophages from the intraperitoneal cavity. The murine peritoneal macrophages in PBS were collected and centrifuge at 450x g for 5 mins at 4°C. One ml of Red blood cell lysing buffer Hybri-Max (Sigma-Aldrich) was added to the cell pellet and resuspended for 3 mins. Fourteen ml of PBS was added and the tube was centrifuged at 450x g for 5 mins at 4°C. The cells were washed again with PBS before culturing in RPMI-1640 supplemented with 10% heat-inactivated FCS, 1% penicillin/streptomycin, 1% amphotericin B and 0.5% gentamycin.

### Survival curve of mice after treatment

DV2-Infected or mock-infected wild type or TLR6^*-/-*^ C57BL/6 mice were monitored daily and observed for any abnormal signs which could be symptoms of infection for up to day 21 post-infection. DV NS1 protein-treated or His-tag-treated wild type or TLR6^*-/-*^ C57BL/6 mice were monitored daily and observed for any abnormal signs which could be symptoms of infection for up to day 7 post-infection.

### Statistical analysis

The statistical comparisons were carried out using two tailed Student’s *t*-test for repeated measurements when applicable. The significance level was set at *: p < 0.05, **: p < 0.005, ***: p < 0.0001. Data shown are obtained from three independent experiments unless stated otherwise. Kruskal-Wallis test was used for non-parametric data set. Log-rank test was used to compare the survival curves of mice.

## Supporting Information

S1 Fig Mock-infected and DV2-infected PBMC on day 1 to day 3 post-infection were stained using rabbit anti-TLR6 antibody, goat anti-rabbit DyLight 633 (APC) antibody and mouse anti-CD14 antibody conjugated with FITC. The cell debris was excluded by gating and the stained cells were analyzed using flow cytometry.
(PPTX)

S2 Fig Mock-infected and DV2-infected PBMC on day 3 post-infection were also stained using mouse anti-TLR2 antibody, goat anti-mouse DyLight 633 (APC) antibody and mouse anti-CD14 antibody conjugated with FITC. The cell debris was excluded by gating and the stained cells were analyzed using flow cytometry. The percentages of cell subset population were indicated on the representative results of three independent experiments obtained using separate PBMC from three donors (i, ii & iii).
(PPTX)

S3 Fig Expression of TLR6 on mock-infected (red) and DV2-infected PBMC (green) on day 1 (3A), day 2 (3B) and day 3 (3C) post-infection were assayed using flow cytometry. PBMC were stained using rabbit anti-TLR6 antibody, goat anti-rabbit DyLight 633 (APC) antibody and mouse anti-CD14 antibody conjugated with FITC. CD14+ cells were selected by setting gate to select for FITC positive cells. 15, 000 CD14+ cells were analyzed for each sample and representative results of three independent experiments obtained using separate PBMC from three donors were shown. Expression of TLR6 on CD14- mock-infected (blue) and CD14- DV2-infected (yellow) PBMC on day 3 post-infection were also shown (3C). Expression of TLR2 on mock-infected (Red) and DV2-infected PBMC (Green) on day 1 (3D), day 2 (3E) and day 3 (3F) post-infection were assayed using flow cytometry. PBMC were stained using mouse anti-TLR2 antibody, goat anti-mouse DyLight 633 (APC) antibody and mouse anti-CD14 antibody conjugated with FITC. CD14+ cells were selected by setting gate to select for FITC positive cells. 15, 000 CD14+ cells were analyzed for each sample and representative results of three independent experiments obtained using separate PBMC from three donors were shown. Expression of TLR2 on CD14- mock-infected (blue) and CD14- DV2-infected (yellow) PBMC on day 3 post-infection were also shown (3F).
(PPTX)

S4 Fig TLR2 and TLR6 of PBMC from 3 donors were blocked prior to treatment with ultrapure LPS at 2.5 x 10^4^ EU/ml. IL-6 (4A) and TNF-α (4B) produced by the treated PBMC on day 2 post-treatment were assayed using ELISA. Data represent mean ± SEM of three independent experiments obtained using separate PBMC from three donors.
(PPTX)

S5 Fig Untransfected HEK 293 cells, HEK 293 cells transfected with SEAP reporter plasmid or pDUO-hTLR6/TLR2 plasmid and HEK293 cells transfected with both plasmids were lysed and 15 μg of total protein was loaded into SDS-PAGE gel. The presence of TLR6 protein in the cell lysate was detected using rabbit anti-TLR6 antibody and goat anti-rabbit HRP conjugated antibody on the Western blot (5A). The intensities of the TLR6 bands were normalized against the intensity of the corresponding actin bands and were plotted on the graph (5B). The presence of TLR2 protein in the cell lysate was detected using rabbit anti-TLR2 antibody and goat anti-rabbit HRP conjugated antibody on the Western blot (5C). Actin was used as loading control. The intensities of the TLR2 bands were normalized against the intensity of the corresponding actin bands and were plotted on the graph (5D).
(PPTX)

S1 Table Analysis of TLR2+ and TLR6+ cells of DV2-infected PBMC. Mock-infected (a) and DV2-infected (b) PBMC were harvested at day 3 post-infection and stained using rabbit anti-TLR6 antibody, goat anti-rabbit DyLight 633 (APC) antibody and mouse anti-TLR2 antibody conjugated with FITC. 30, 000 cells were analyzed using flow cytometry and gating was set to omit cell debris. The number of cells in the different regions is expressed as percentage to the total number of cell analyzed. The median of the fluorescence of the cells in the different region is also given. Region 1 (R1) contains cells which are negative for FITC but positive for APC. Region 2 (R2) contains cells which are positive for both FITC and APC. Region 3 (R3) contains cells which are negative for both FITC and APC. Region 4 (R4) contains cells which are positive for FITC but negative for APC. Region 5 (R2 + R4) contains cells which express TLR2. Region 6 (R1 + R2) contains cells which express TLR6. Each table shows the results obtained from PBMC of one donor. PBMC of table (ii) contain 3.84% CD3+CD20+ cells while PBMC of table (iii) contain 3.94% CD3+CD20+ cells.
(DOCX)
