# Supplementary figures and images for: Activation of TLR2 and TLR6 by Dengue NS1 Protein and Its Implications in the Immunopathogenesis of Dengue Virus Infection
Source: PLoS Pathog. 2015 Jul 30;11(7):e1005053. doi: 10.1371/journal.ppat.1005053 (PMC4520596; doi:10.1371/journal.ppat.1005053)

## Slide 1
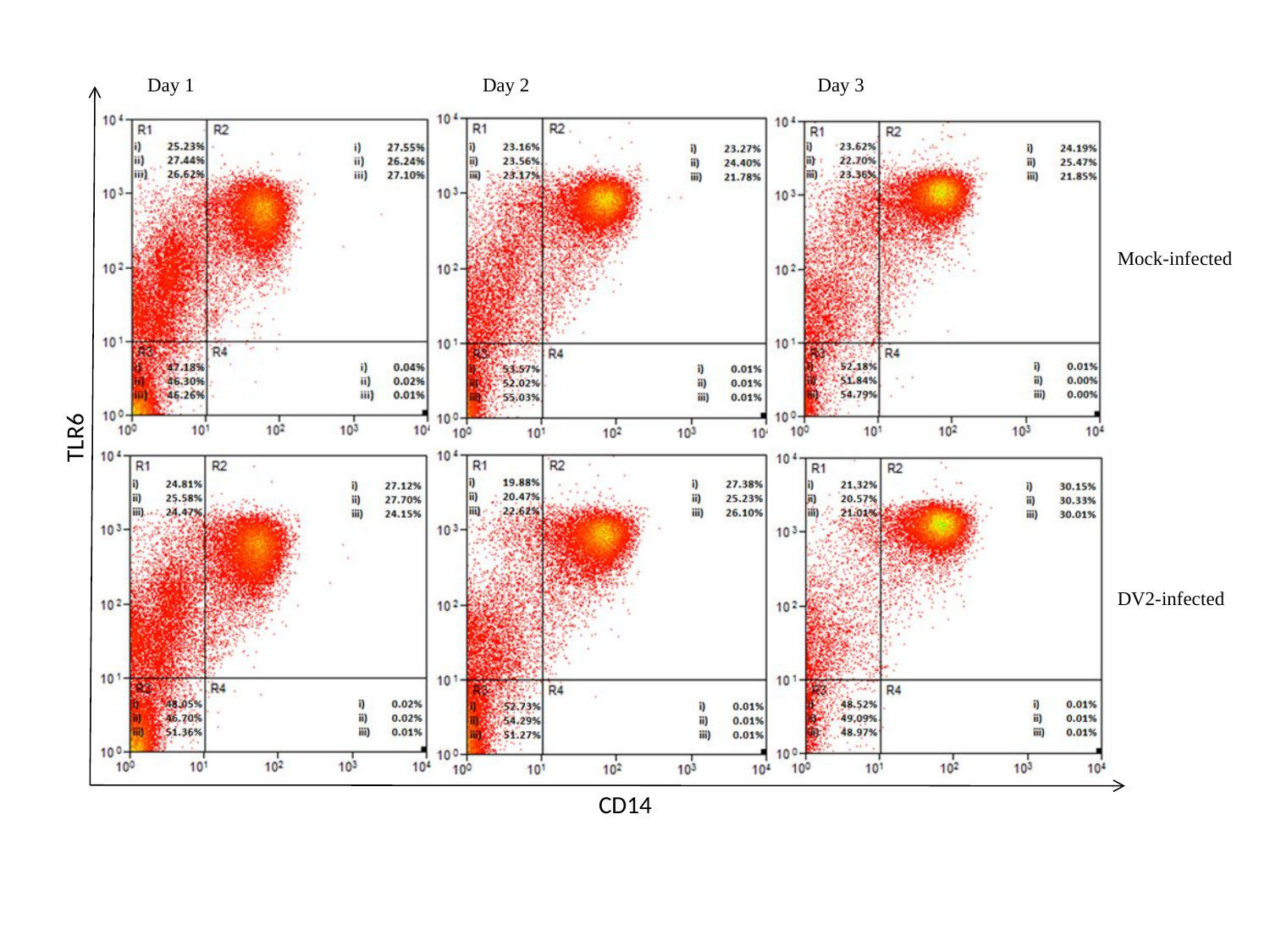

Day 1
Day 2
Day 3
Mock-infected
 TLR6
DV2-infected
 CD14

Supplement: S1 Fig — The cell debris was excluded by gating and the stained cells were analyzed using flow cytometry. (PPTX) [file ppat.1005053.s001.pptx]

## Slide 1
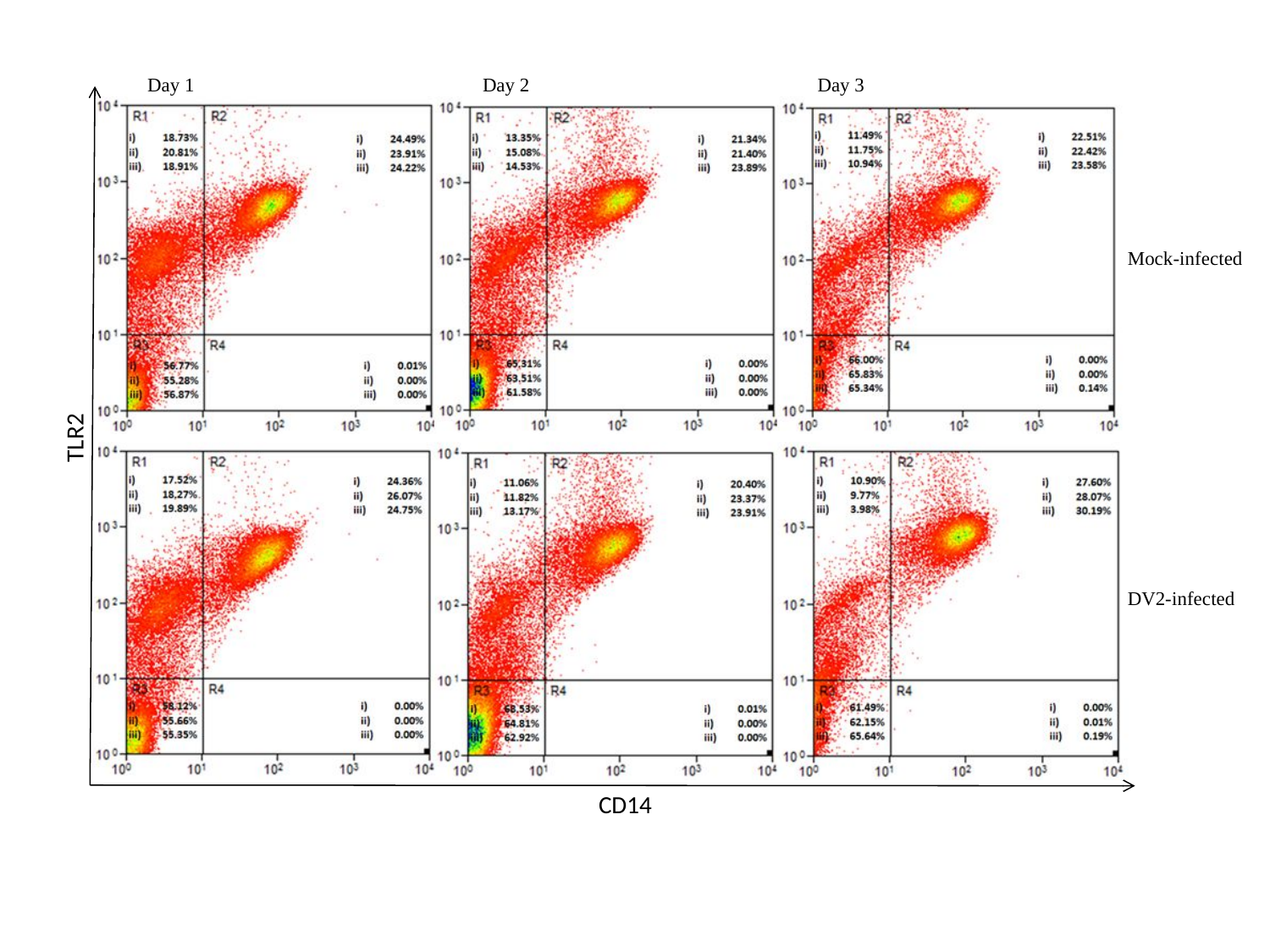

Day 1
Day 2
Day 3
Mock-infected
 TLR2
DV2-infected
 CD14

Supplement: S2 Fig — The cell debris was excluded by gating and the stained cells were analyzed using flow cytometry. The percentages of cell subset population were indicated on the representative results of three independent experiments obtained using separate PBMC from three donors (i, ii & iii). (PPTX) [file ppat.1005053.s002.pptx]

## Slide 1
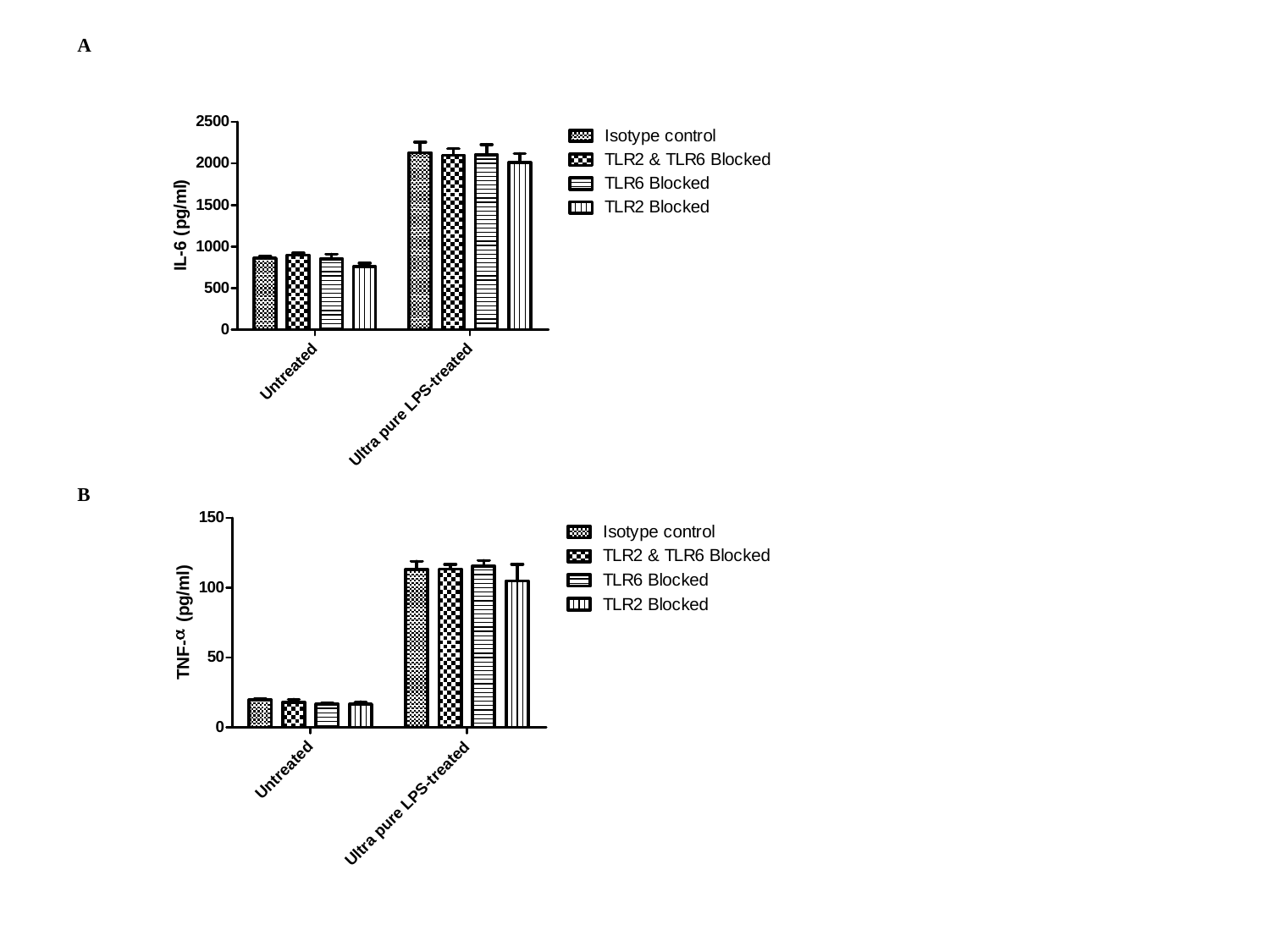

A
B

Supplement: S4 Fig — IL-6 (4A) and TNF-α (4B) produced by the treated PBMC on day 2 post-treatment were assayed using ELISA. Data represent mean ± SEM of three independent experiments obtained using separate PBMC from three donors. (PPTX) [file ppat.1005053.s004.pptx]

## Slide 1
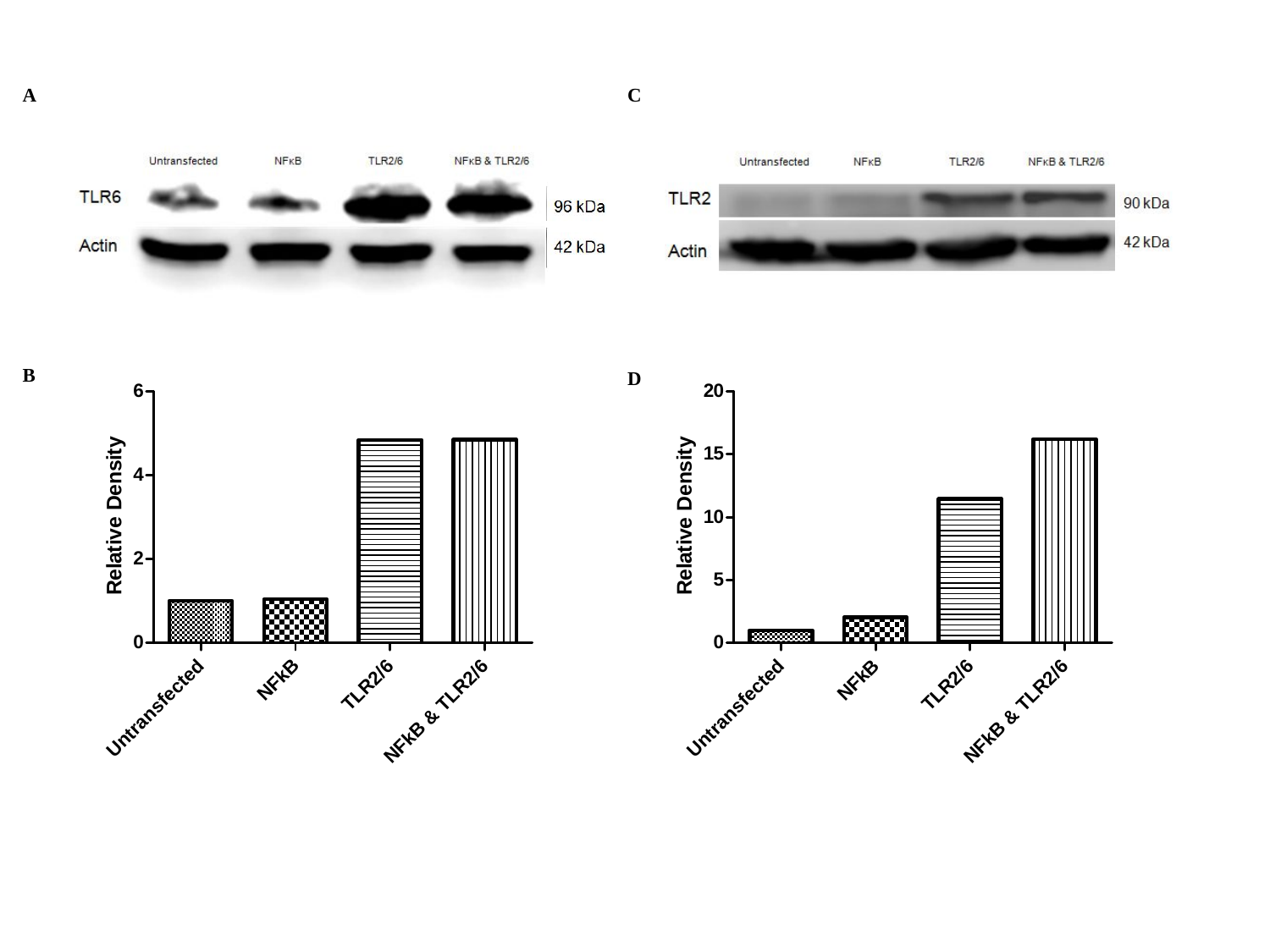

A
C
B
D

Supplement: S5 Fig — The presence of TLR6 protein in the cell lysate was detected using rabbit anti-TLR6 antibody and goat anti-rabbit HRP conjugated antibody on the Western blot (5A). The intensities of the TLR6 bands were normalized against the intensity of the corresponding actin bands and were plotted on the graph (5B). The presence of TLR2 protein in the cell lysate was detected using rabbit anti-TLR2 antibody and goat anti-rabbit HRP conjugated antibody on the Western blot (5C). Actin was used as loading control. The intensities of the TLR2 bands were normalized against the intensity of the corresponding actin bands and were plotted on the graph (5D). (PPTX) [file ppat.1005053.s005.pptx]
