# Supplementary material for: Activation of TLR2 and TLR6 by Dengue NS1 Protein and Its Implications in the Immunopathogenesis of Dengue Virus Infection
Source: PLoS Pathog. 2015 Jul 30;11(7):e1005053. doi: 10.1371/journal.ppat.1005053 (PMC4520596; doi:10.1371/journal.ppat.1005053)
Supplement: S3 Fig — PBMC were stained using rabbit anti-TLR6 antibody, goat anti-rabbit DyLight 633 (APC) antibody and mouse anti-CD14 antibody conjugated with FITC. CD14+ cells were selected by setting gate to select for FITC positive cells. 15, 000 CD14+ cells were analyzed for each sample and representative results of three independent experiments obtained using separate PBMC from three donors were shown. Expression of TLR6 on CD14- mock-infected (blue) and CD14- DV2-infected (yellow) PBMC on day 3 post-infection were also shown (3C). Expression of TLR2 on mock-infected (Red) and DV2-infected PBMC (Green) on day 1 (3D), day 2 (3E) and day 3 (3F) post-infection were assayed using flow cytometry. PBMC were stained using mouse anti-TLR2 antibody, goat anti-mouse DyLight 633 (APC) antibody and mouse anti-CD14 antibody conjugated with FITC. CD14+ cells were selected by setting gate to select for FITC positive cells. 15, 000 CD14+ cells were analyzed for each sample and representative results of three independent experiments obtained using separate PBMC from three donors were shown. Expression of TLR2 on CD14- mock-infected (blue) and CD14- DV2-infected (yellow) PBMC on day 3 post-infection were also shown (3F). (PPTX) [file ppat.1005053.s003.pptx]

## Slide 1
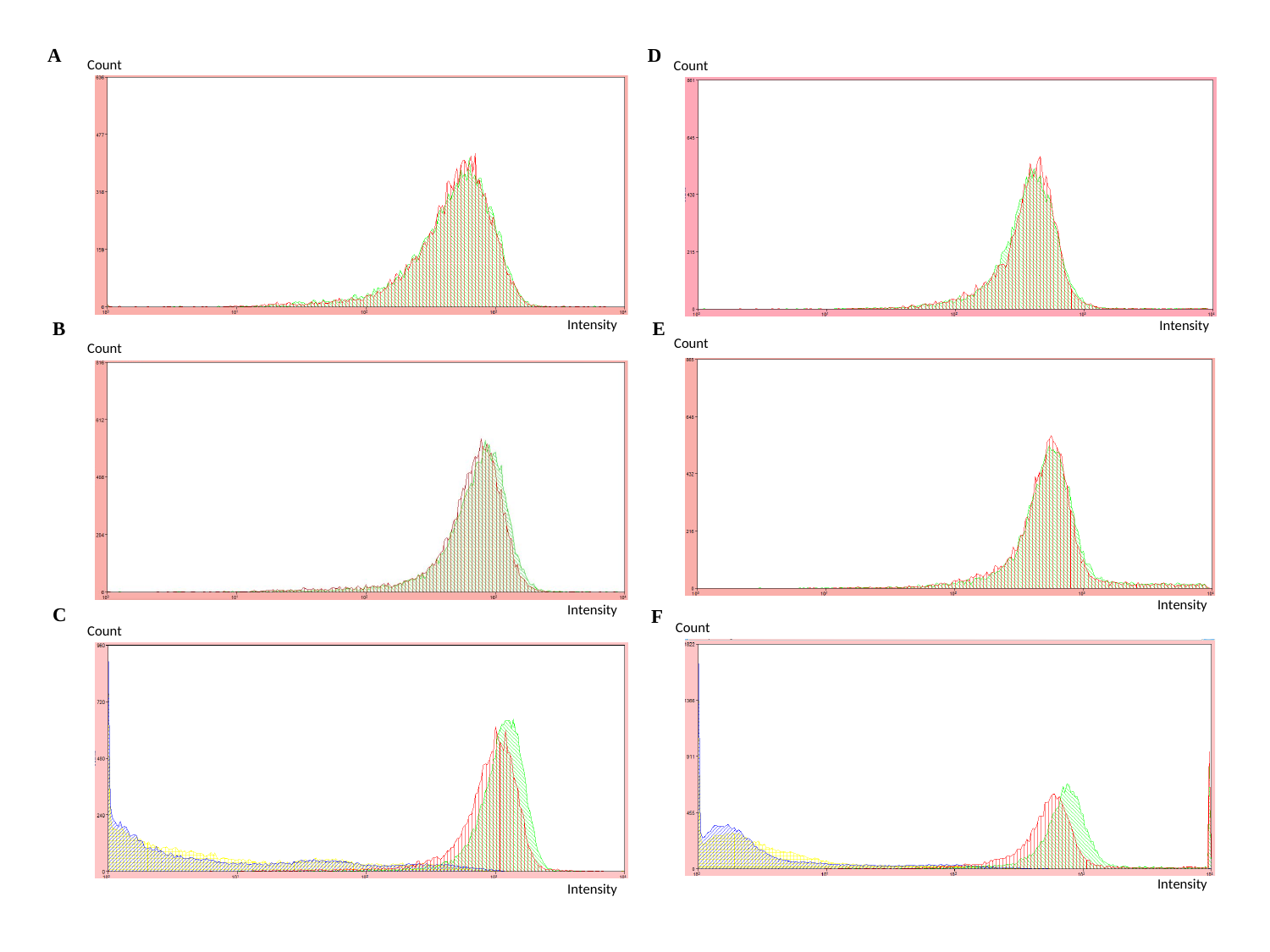

A
D
Count
Count
Intensity
B
E
Intensity
Count
Count
Intensity
Intensity
C
F
Count
Count
Intensity
Intensity
