# Supplementary material for: Activation of TLR2 and TLR6 by Dengue NS1 Protein and Its Implications in the Immunopathogenesis of Dengue Virus Infection
Source: PLoS Pathog. 2015 Jul 30;11(7):e1005053. doi: 10.1371/journal.ppat.1005053 (PMC4520596; doi:10.1371/journal.ppat.1005053)
Supplement: S1 Table — Mock-infected (a) and DV2-infected (b) PBMC were harvested at day 3 post-infection and stained using rabbit anti-TLR6 antibody, goat anti-rabbit DyLight 633 (APC) antibody and mouse anti-TLR2 antibody conjugated with FITC. 30, 000 cells were analyzed using flow cytometry and gating was set to omit cell debris. The number of cells in the different regions is expressed as percentage to the total number of cell analyzed. The median of the fluorescence of the cells in the different region is also given. Region 1 (R1) contains cells which are negative for FITC but positive for APC. Region 2 (R2) contains cells which are positive for both FITC and APC. Region 3 (R3) contains cells which are negative for both FITC and APC. Region 4 (R4) contains cells which are positive for FITC but negative for APC. Region 5 (R2 + R4) contains cells which express TLR2. Region 6 (R1 + R2) contains cells which express TLR6. Each table shows the results obtained from PBMC of one donor. PBMC of table (ii) contain 3.84% CD3+CD20+ cells while PBMC of table (iii) contain 3.94% CD3+CD20+ cells. (DOCX) [file ppat.1005053.s006.docx]

Supplementary Tables:

**Table 1:** **Analysis of TLR2+ and TLR6+ cells of DV2-infected PBMC.**

Mock-infected (a) and DV2-infected (b) PBMC were harvested at day 3 post-infection and stained using rabbit anti-TLR6 antibody, goat anti-rabbit DyLight 633 (APC) antibody and mouse anti-TLR2 antibody conjugated with FITC. 30, 000 cells were analyzed using flow cytometry and gating was set to omit cell debris. The number of cells in the different regions is expressed as percentage to the total number of cell analyzed. The median of the fluorescence of the cells in the different region is also given. Region 1 (R1) contains cells which are negative for FITC but positive for APC. Region 2 (R2) contains cells which are positive for both FITC and APC. Region 3 (R3) contains cells which are negative for both FITC and APC. Region 4 (R4) contains cells which are positive for FITC but negative for APC. Region 5 (R2 + R4) contains cells which express TLR2. Region 6 (R1 + R2) contains cells which express TLR6. Each table shows the results obtained from PBMC of one donor. PBMC of table (ii) contain 3.84% CD3+CD20+ cells while PBMC of table (iii) contain 3.94% CD3+CD20+ cells.

(i)

| Treatment | Mock-infected | | | | DV2-infected | | | |
| --- | --- | --- | --- | --- | --- | --- | --- | --- |
| Region | Median  (FITC) | Median  (APC) | Count | Population % | Median  (FITC) | Median  (APC) | Count | Population % |
| Total | 5.46 | 5.08 | 30000 | 100.00 | 7.03 | 5.46 | 30000 | 100.00 |
| R1 | 3.29 | 21.54 | 1727 | 5.76 | 7.84 | 21.54 | 432 | 1.44 |
| R2 | 28.76 | 27.74 | 8858 | 29.53 | 35.72 | 46.00 | 12334 | 41.11 |
| R3 | 1.60 | 1.44 | 17108 | 57.03 | 1.16 | 1.04 | 15535 | 51.78 |
| R4 | 15.57 | 7.03 | 2307 | 7.69 | 16.14 | 7.03 | 1699 | 5.66 |
| R5 (R2+R4) | 24.89 | 20.78 | 11165 | 37.22 | 32.05 | 37.04 | 14033 | 46.78 |
| R6  (R1+R2) | 25.81 | 26.76 | 10585 | 35.28 | 34.46 | 46.00 | 12766 | 42.55 |

(ii)

| Treatment | Mock-infected | | | | DV2-infected | | | |
| --- | --- | --- | --- | --- | --- | --- | --- | --- |
| Region | Median  (FITC) | Median  (APC) | Count | Population % | Median  (FITC) | Median  (APC) | Count | Population % |
| Total | 4.56 | 8.12 | 30000 | 100 | 10.09 | 44.37 | 30000 | 100 |
| R1 | 5.66 | 38.4 | 7228 | 24.09 | 5.27 | 38.4 | 3074 | 10.25 |
| R2 | 20.04 | 63.67 | 7460 | 24.87 | 24.89 | 162.84 | 14001 | 46.67 |
| R3 | 2.85 | 1 | 14435 | 48.12 | 2.65 | 1 | 12132 | 40.44 |
| R4 | 17.35 | 3.81 | 877 | 2.92 | 24.01 | 3.54 | 793 | 2.64 |
| R5 (R2+R4) | 19.33 | 57.13 | 8337 | 27.79 | 24.89 | 157.06 | 14794 | 49.31 |
| R6  (R1+R2) | 10.85 | 49.44 | 14688 | 48.96 | 22.34 | 146.12 | 17075 | 56.92 |

(iii)

| Treatment | Mock-infected | | | | DV2-infected | | | |
| --- | --- | --- | --- | --- | --- | --- | --- | --- |
| Region | Median  (FITC) | Median  (APC) | Count | Population % | Median  (FITC) | Median  (APC) | Count | Population % |
| Total | 6.78 | 1.54 | 30000 | 100 | 9.05 | 1.6 | 30000 | 100 |
| R1 | 9.05 | 29.82 | 1712 | 5.71 | 8.42 | 27.74 | 949 | 3.16 |
| R2 | 17.35 | 37.04 | 8533 | 28.44 | 34.46 | 68.44 | 9965 | 33.22 |
| R3 | 4.24 | 1 | 17828 | 59.43 | 4.56 | 1 | 15890 | 52.97 |
| R4 | 14.48 | 1 | 1927 | 6.42 | 14.48 | 1 | 3196 | 10.65 |
| R5 (R2+R4) | 16.73 | 29.82 | 10460 | 34.87 | 24.89 | 39.81 | 13161 | 43.87 |
| R6  (R1+R2) | 15.57 | 35.72 | 10245 | 34.15 | 30.92 | 63.67 | 10914 | 36.38 |
